# DAF-18/PTEN inhibits germline zygotic gene activation during primordial germ cell quiescence

**DOI:** 10.1371/journal.pgen.1009650

**Published:** 2021-07-21

**Authors:** Amanda L. Fry, Amy K. Webster, Julia Burnett, Rojin Chitrakar, L. Ryan Baugh, E. Jane Albert Hubbard

**Affiliations:** 1 Skirball Institute of Biomolecular Medicine, Department of Cell Biology, NYU Grossman School of Medicine, New York, New York, United States of America; 2 Department of Biology, Center for Genomic and Computational Biology, Duke University, Durham, North Carolina, United States of America; University of Cambridge, UNITED KINGDOM

## Abstract

Quiescence, an actively-maintained reversible state of cell cycle arrest, is not well understood. PTEN is one of the most frequently lost tumor suppressors in human cancers and regulates quiescence of stem cells and cancer cells. The sole PTEN ortholog in *Caenorhabditis elegans* is *daf-18*. In a *C*. *elegans* loss-of-function mutant for *daf-18*, primordial germ cells (PGCs) divide inappropriately in L1 larvae hatched into starvation conditions, in a TOR-dependent manner. Here, we further investigated the role of *daf-18* in maintaining PGC quiescence in L1 starvation. We found that maternal or zygotic *daf-18* is sufficient to maintain cell cycle quiescence, that *daf-18* acts in the germ line and soma, and that *daf-18* affects timing of PGC divisions in fed animals. Importantly, our results also implicate *daf-18* in repression of germline zygotic gene activation, though not in germline fate specification. However, TOR is less important to germline zygotic gene expression, suggesting that in the absence of food, *daf-18*/PTEN prevents inappropriate germline zygotic gene activation and cell division by distinct mechanisms.

## Author summary

PTEN is the second-most commonly lost tumor suppressor in human cancers. The *C*. *elegans daf-18* gene encodes the worm version of PTEN. In the *C*. *elegans* embryo, the two primordial germ cells (Z2 and Z3) are born, and they will eventually form the entire germ line. These two cells are quiescent in the embryo. They do not divide until after the L1 larva hatches and starts feeding. That is, if the L1 larva hatches in the absence of food, these cells will remain quiescent until the worm feeds. Previous studies showed that in worms that have lost *daf-18* function, Z2 and Z3 inappropriately begin dividing in the L1 larva even in the absence of food. We found that *daf-18* normally prevents this inappropriate division when *daf-18* function is provided from the mother or from within the larva, and when it is provided from the germ cells or from the somatic cells. We also found that without *daf-18*, certain germline genes are inappropriately expressed. Finally, we found that one of the mechanisms known to regulate cell division in this context does not similarly regulate germline gene activity.

## Introduction

Cell cycle quiescence is an actively maintained state of non-proliferation. The best characterized quiescent state, known as “G_0_,” is associated with exit from the cell cycle after M phase. Depending on the cell type, arrested cells adopt a variety of altered metabolic states while they await an activation cue, usually a growth factor signal [1–3]. Response to these cues ultimately leads to post-translational modifications of cell cycle proteins, returning the cell to a cycle of continuous G1, S, G2 and M phases [4].

Cell cycle quiescence can also occur in the G2 phase of the cell cycle. Though far less well-understood, G2 quiescence has been observed in vertebrate muscle stem cells, *Drosophila* neural stem cells, and *C*. *elegans* germ cells [5–7]. Proper regulation of quiescence is particularly important in founder cells such as stem cells [8]. Inappropriate quiescence of these cells can cause loss of downstream differentiated cell populations, aberrant tissue homeostasis, and failure to repair tissue damage. Inappropriate exit of these cells from quiescence can prematurely deplete the stem cell pool and similarly cause defects in tissue homeostasis. Thus, understanding how quiescence is modulated is crucial for understanding the behavior of stem cells *in vivo*. In addition, this understanding is important for the development of new therapies to target cancer stem-like cells since quiescent cancer stem cells are refractory to common chemotherapies and are likely responsible for recurrent cancer [1,9–11].

The regulation of both *Drosophila* neural stem cell and *C*. *elegans* germ cell quiescence has been linked to organismal nutrient status by insulin-PI3-kinase (PI3K) signaling and PTEN. Phosphatase and Tensin homolog (PTEN) regulates nutrient-sensitive pathways and inhibits proliferation of stem/progenitor cells [12–17], cancer cells [18], and cancer stem cell-like populations [19]. PTEN is the second-most commonly lost tumor suppressor in human cancers [12,20]. Molecularly, PTEN functions as a lipid and protein phosphatase [18]. It dephosphorylates the lipid second messenger phosphatidylinositol _(3,4,5)_ tri-phosphate (PIP_3_), converting it to PI_(4,5)_P (PIP_2_). Thus, PTEN opposes the activity of PI3K, diminishing the effects of upstream receptors (such as growth factor receptors and the insulin receptor) on proteins activated by PIP_3_, such as AKT, a positive effector of cell cycle progression [21]. Interestingly, PTEN loss in mammals can render tumor cells insensitive to dietary restriction [22,23], consistent with PTEN mediating nutritional control of cell proliferation. The breadth of mechanisms by which PTEN regulates cell quiescence is not fully understood. Understanding the mechanisms by which PTEN regulates G2 stem cell quiescence in the context of normal development may also uncover additional roles important for cancer.

The sole PTEN ortholog in *C*. *elegans* is *daf-18*, and it was the first PTEN homolog to be described in a genetically tractable model organism. It was identified as a negative regulator of insulin signaling in dauer formation (dauer is a stress-resistant larval stage; “daf” stands for “abnormal DAuer Formation”) [24–26]. In *C*. *elegans*, the two primordial germ cells (PGCs) are born relatively early in embryogenesis but do not divide again until worms have hatched and begin feeding. Loss of *daf-18* causes G2-arrested PGCs to proliferate inappropriately in first larval stage worms (L1) despite starvation [5]. Loss of *daf-18* also interferes with later germ cell cycle arrest during dauer [27] via non-autonomous activity in the somatic gonad [28]. Remarkably, human PTEN can rescue *daf-18* mutant dauer and longevity phenotypes [29], indicating conserved function.

The current model for regulation of PGC cell cycle by DAF-18 in starved L1 worms is that DAF-18 regulation occurs downstream of insulin signaling, through PI3K, Akt and TOR, but is independent of DAF-16 FOXO [5,30–34]. While *C*. *elegans* germ cells arrest in the G2 phase of the cell cycle during L1 starvation [5], most somatic cells arrest in G1 in a *daf-16*-dependent fashion through regulation of the cyclin dependent kinase inhibitor CKI-1 [30,35].

To further understand how DAF-18 PTEN acts to maintain L1 PGC quiescence, we took advantage of a null allele *daf-18(ok480)* and a live marker for germ cells. Our results suggest that DAF-18 influences the onset of germ cell division in both starved and fed L1 larvae, and that DAF-18 appears to act in both the germ line and the soma to regulate PGC quiescence. We also determined that either maternal or zygotically supplied DAF-18 can maintain quiescence. Finally, we investigated the relationship between germline zygotic gene activation and PGC quiescence. We discovered that DAF-18 maintains transcriptional quiescence in addition to cell division quiescence. However, we found that transcriptional quiescence is not as sensitive to TOR as is cell cycle quiescence, suggesting that an alternative effector may contribute to transcriptional quiescence downstream of DAF-18.

## Results

### DAF-18 influences the timing of PGC division onset in both starved and fed L1 larvae

**Fig 1:**
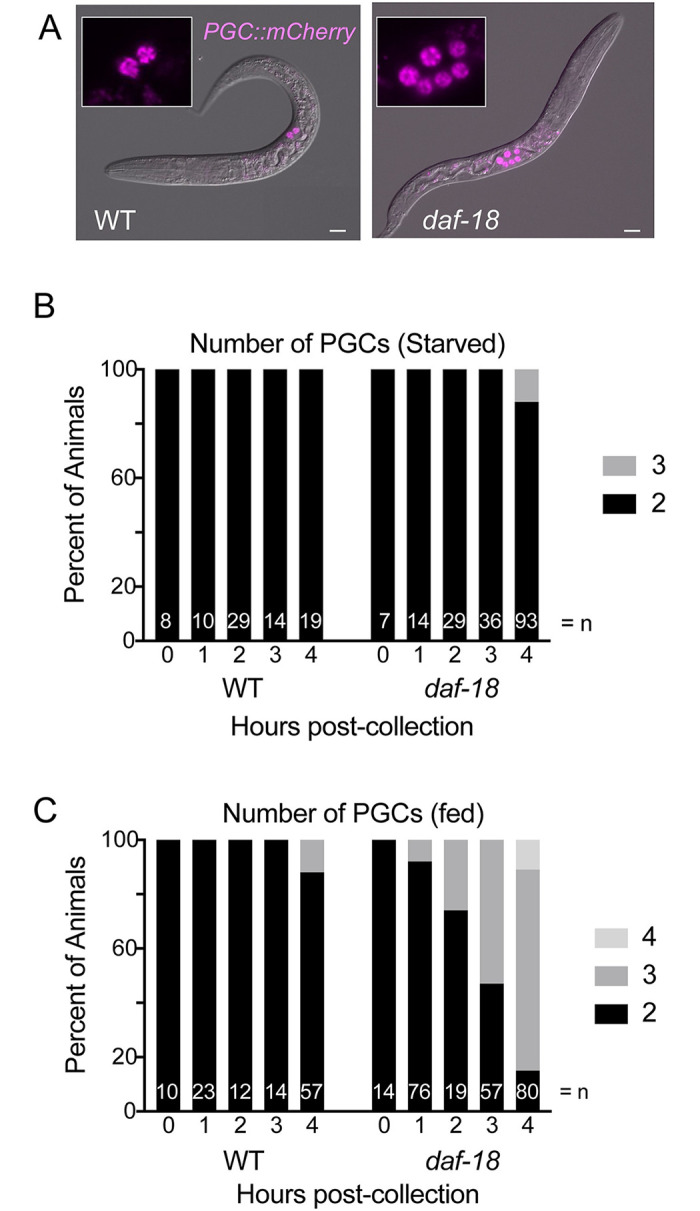
DAF-18 promotes PGC quiescence in both fed and starved larvae. (A) Wild type (WT) and *daf-18(ok480)* mutant L1 larvae, starved for 3 days after hatching, show 2 or an average of 6 primordial germ cells (PGCs), respectively. The marker *PGC*::*mCherry* (*naSi2*) is expressed in the PGCs (and in ~10% of animals, expression is seen in one additional cell in the head). Scale bar represents 10μm. (B) and (C), Animals were synchronized within 2 hours of hatching and monitored for PGC divisions during starvation (B) and in the presence of food (C). Summary of 2 replicate experiments.

The primordial germ cells (PGCs) Z2 and Z3 are born by division of the P4 cell in the embryo and do not divide further until L1 larvae have hatched and begun feeding. In worms bearing loss-of-function mutations in *daf-18*, PGCs divide in L1 larvae in the absence of food [5]. We wished to determine when PGCs start dividing in *daf-18* mutants. We examined live late-stage embryos (3-fold) in a strain bearing a P-granule marker (see Methods) and found that virtually all *daf-18* mutant embryos (542/543 embryos) had the normal two PGCs. To determine when PGCs of starved *daf-18* mutants first divide relative to fed wild-type controls, we performed a time-course analysis in worms carrying *naSi2*, a germline-expressed transgene of mCherry fused to histone H2B [36] (Fig 1A). We first established that *daf-18* mutant embryos collected at the 2–4 cell stage hatch at the same time as wild-type embryos (S1 Fig). We then monitored PGC divisions in L1 larvae that had been tightly synchronized relative to hatching (see Methods) in wild-type and *daf-18* mutants in the absence and presence of food. We found that PGCs in starved *daf-18* mutants began to divide at the same time (4 hours post-collection, see Methods) as did fed wild-type worms (Fig 1B and 1C). Surprisingly, fed *daf-18* mutant PGCs divide earlier than fed wild-type L1 larvae (Fig 1C). Thus, PGCs divide several hours earlier in fed *daf-18* mutants than starved *daf-18* mutants. These results indicate that while *daf-18* mutants show food-independent PGC division that mimics the timing of PGC division in fed wild-type worms, PGCs in *daf-18* mutants also appear primed to divide such that they initiate divisions early in the presence of food.

### DAF-18 appears to act in both the soma and germ line to regulate PGC quiescence

**Fig 2:**
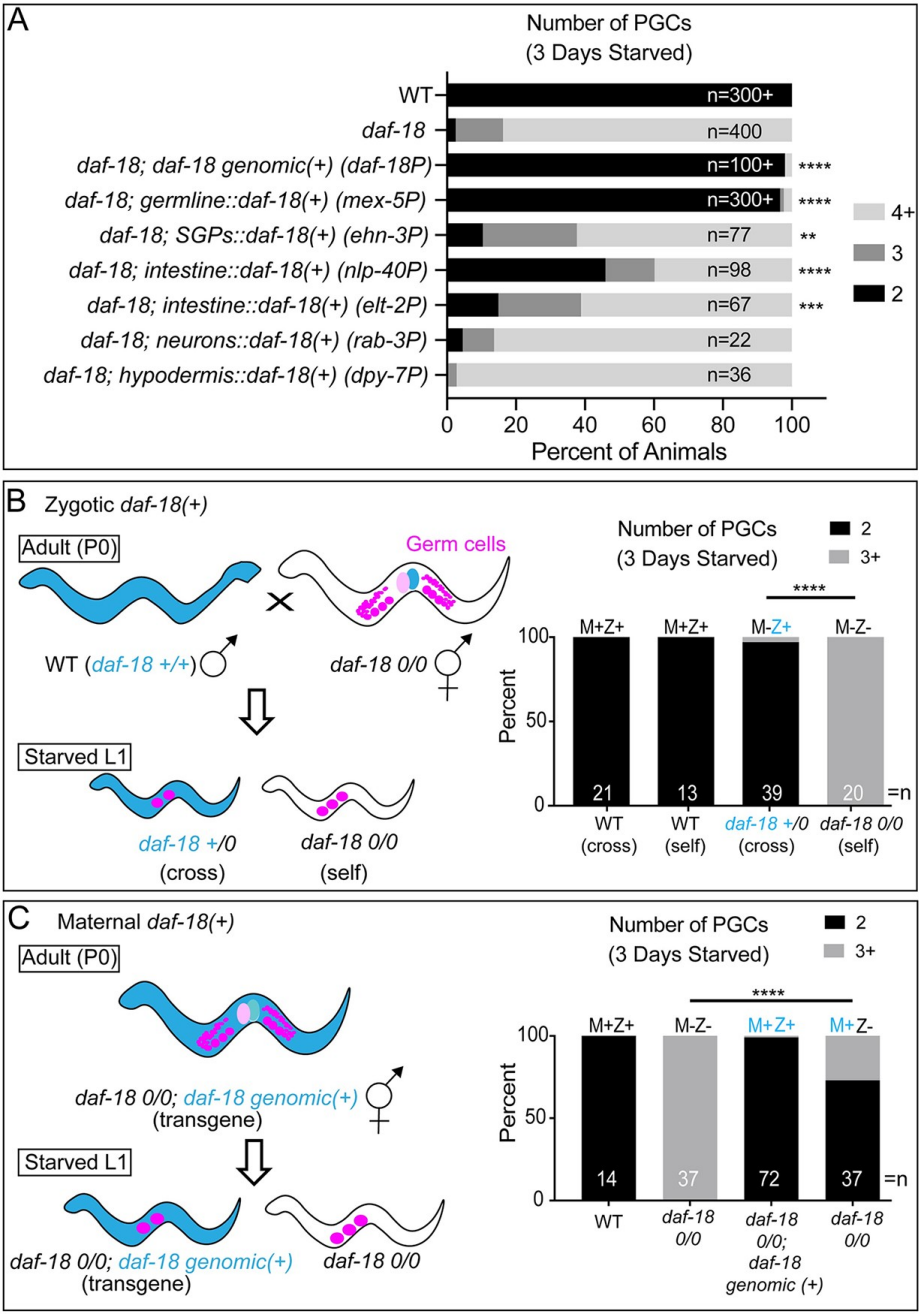
DAF-18 acts in the germ line and soma to suppress PGC division during starvation. (A) The number of PGCs in L1 larvae was assessed after 3 days of starvation. Genotypes indicate the promoters used to drive tissue-restricted expression of the *daf-18* coding region containing all introns and *daf-18* 3’UTR, with the exception of *germline*::*daf-18(+)*, which contains the same *daf-18* coding region with introns, but the *nos-2* 3’UTR. All animals carry *PGC*::*mCherry*, except for *daf-18; germline*::*daf-18(+)*, which carries the PGC marker *glh-1*::*GFP* or no additional PGC marker (*germline*::*daf-18* is tagged with GFP; see S2 Fig). n values display the total number of animals examined for each genotype. Chi-square statistical tests (or two-sided Fisher’s exact test for *ehn-3P*, *elt-2P*, *rab-3P and dpy-7P*) were performed on each genotype compared to its own experimental control group (*daf-18(ok480)* mutant with *PGC*::*mCherry*) and significance is displayed: **p<0.01, ***p<0.001, ****p<0.0001. Additional independently-generated transgenic lines were examined for several transgenes, with similar results found: 3 additional lines of *daf-18P*::*daf-18(+)* (suppressed *daf-18*), 2 additional lines of *nlp-40P*::*daf-18(+)* (suppressed *daf-18*), 1 additional line of *dpy-7P*::*daf-18(+)* (no suppression), and 1 additional line of *rab-3P*::*daf-18(+)* (no suppression). (B) Zygotic DAF-18 maintains PGC quiescence. Blue color indicates the presence of *daf-18(+)*. Strategy is shown to generate Maternal-Zygotic+ (M-Z+) by crossing paternal *daf-18(+)* to *daf-18* mutant hermaphrodites. This strategy gives starved L1 cross-progeny one wild-type copy of *daf-18* in the absence of maternal *daf-18*. Cross- versus self-progeny were identified by the presence/absence of a bright somatic fluorescent marker from the paternal P0 strain (carrying *cdc-42*::*GFP*). Number of PGCs were assessed after 3 days of starvation of L1 progeny. Statistical significance determined by two-sided Fisher’s exact test. ****p<0.0001. (C) Maternal DAF-18 also maintains PGC quiescence. Blue color indicates the presence of *daf-18(+)*. Strategy is shown to provide maternal *daf-18(+)* in the presence (M+Z+) or absence (M+Z-) of zygotic *daf-18(+)* by analyzing the progeny of *daf-18* mutants (0/0) carrying a *daf-18(+)* genomic transgene. This transgene was stochastically lost in progeny, yielding M+Z+ or M+Z- L1 larvae, which were assessed for PGC numbers after 3 days of starvation. Statistical significance determined by two-sided Fisher’s exact test. ****p<0.0001.

Previous studies found that transgenic expression of *daf-18(+)* genomic sequences restores PGC quiescence to *daf-18* mutants [32]. These transgenes were expressed from extrachromosomal arrays, a technique which often precludes germline expression [37]. We generated a similar transgene bearing the *daf-18(+)* genomic sequence on an extrachromosomal array, with a trans-splice to GFP::H2B to follow expression without altering the DAF-18 protein itself (see Methods). We confirmed that PGC quiescence was fully restored in animals bearing this *daf-18(+)* transgene (Fig 2, “*daf-18 genomic(+)*”). Consistent with other studies showing expression of *daf-18* reporters in varied tissues [38–41], we observed widespread expression of GFP, including within the intestine, neurons, and hypodermis. We did not observe expression from our *daf-18(+)* genomic transgene in the PGCs (S2 Fig).

To determine whether *daf-18(+)* expression from specific somatic tissues might also restore PGC quiescence, we generated plasmids driving *daf-18(+)* expression (similar to genomic array above, including all introns and trans-spliced GFP::H2B, but with alternative sequences 5’ to the *daf-18* ATG) from various tissue-restricted promoters. We found that *daf-18(+)* expression in the intestine or somatic gonad precursors, but not in the hypodermis or neurons, partially restored PGC quiescence in *daf-18* mutants, albeit not to the same level as the genomic array (Fig 2A).

Since *daf-18* is reported to be highly expressed in the germ cells [38,41,42], and regulates L1 PGC division [5], we tested whether germline-expressed *daf-18(+)* can support PGC quiescence in the absence of food. We expressed *daf-18(+)* in the PGCs using a transgene bearing a construct consisting of a germline promoter (*mex-5P*) and 3’ UTR (*nos-2*) flanking the *daf-18* coding region, trans-spliced to GFP (fused to a plextrin homology (PH) domain, which enhanced membrane localization). DNA bearing these sequences was inserted into the genome using CRISPR/Cas9 [43]. These regulatory regions limit expression to the germ line, as seen with our *PGC*::*mCherry* marker using the same regulatory regions (Fig 1). Additionally, the *nos-2* 3’ UTR represses translation of maternal transcripts [44], so expression of DAF-18 from this transgene is likely limited to the zygotic germ line (see S2 Fig and legend for additional information about this transgene). We found this transgene to be highly expressed in the PGCs and, in contrast to partial rescue from *daf-18(+)* driven from intestine and somatic gonad precursor promoters, it nearly fully restored PGC quiescence to *daf-18* mutants (Fig 2A).

Taken together, our results suggest that germline DAF-18 plays a major role in regulating PGC quiescence, but that DAF-18 in somatic tissues (including intestine and somatic gonad precursors, and/or multiple somatic tissues or other tissues we did not test individually) also contributes to PGC quiescence. GFP expression was observed from the genomic and all somatic transgenes in the expected tissues and none was observed in the germ line (S2 Fig). None of the genes from which we chose regulatory regions for somatic tissue-restriction drive high levels of transcription (>100 transcripts per million; see S2 Fig) in the germ line based on single cell RNA sequencing [41,45]. Nevertheless, it remains formally possible that somatic promoter arrays express *daf-18(+)* in the germ line below the level of detection and that this could contribute to the rescue observed with these transgenes.

To investigate DAF-18 functional contributions from the germ line and soma in another way, we targeted *daf-18* by RNAi in wild type, *rrf-1(pk1417)* or *ppw-1(pk2505)* mutants. The *rrf-1* mutant maintains RNAi proficiency in the germ line [46], as well as the intestine and epidermis at least in later stages [47], while the *ppw-1* mutant is defective in germline RNAi, but supports RNAi in somatic cells [48]. We found that *daf-18* RNAi in either the *rrf-1* mutant or in the *rrf-1(+)* wild type efficiently generated the *daf-18* mutant phenotype with >50% penetrance of inappropriate PGC divisions in starved L1 larvae (S3 Fig). By contrast, only 4% of worms subject to *daf-18* RNAi in the *ppw-1* mutant background displayed inappropriate PGC divisions (S3 Fig). Thus, taken together with the results of our heterologous expression analysis, these results are consistent with *daf-18* playing a major role in the germ line to promote PGC quiescence, with an additional role in the intestine and/or other somatic tissues.

### Maternal or zygotic *daf-18* activity maintains PGC quiescence in starved L1 larvae

PGC divisions normally occur early in larval development, a time when maternal or zygotic *daf-18* could regulate PGC divisions. To determine whether zygotic *daf-18(+)* alone is sufficient to confer quiescence, we crossed *daf-18(0)* mutant hermaphrodites with *daf-18(+)* males to generate *daf-18* heterozygous progeny lacking maternal *daf-18(+)* but expressing zygotic *daf-18(+)* (M-Z+) and inspected them for PGC quiescence during L1 starvation. We found that PGC quiescence was restored, indicating that zygotic *daf-18(+)* is sufficient for normal PGC quiescence (Fig 2B).

We then tested whether maternal *daf-18(+)* alone could also maintain PGC quiescence. We examined progeny from *daf-18(0)* mothers carrying a *daf-18(+)* genomic rescuing array (described above). Progeny that had lost the *daf-18(+)* array (see Methods) nevertheless showed PGCs arrested appropriately during starvation (Fig 2C), suggesting that maternal *daf-18(+)* is also sufficient for PGC quiescence.

### Marks of PGC transcriptional activation are elevated in starved *daf-18* mutants prior to cell division

Several marks of active transcription in the PGCs are thought to be dependent on exposure to food (H3K4me2, H3K4me3, and “active” Pol II). The levels of these marks are reported to be low or absent in PGCs of late-stage embryos or starved L1 larvae relative to PGCs of fed L1 larvae or relative to somatic cells [49–52]. Specifically, the levels of H3K4me2 detection in PGCs of L1 larvae is low relative to somatic cells prior to feeding and is elevated after feeding and prior to cell division [52]. In addition, “active” Pol II (phosphorylated Ser2 on the C terminal domain of Pol II; P-Ser2) is more readily detected in germ cells once L1 larvae begin to feed [49].

**Fig 3:**
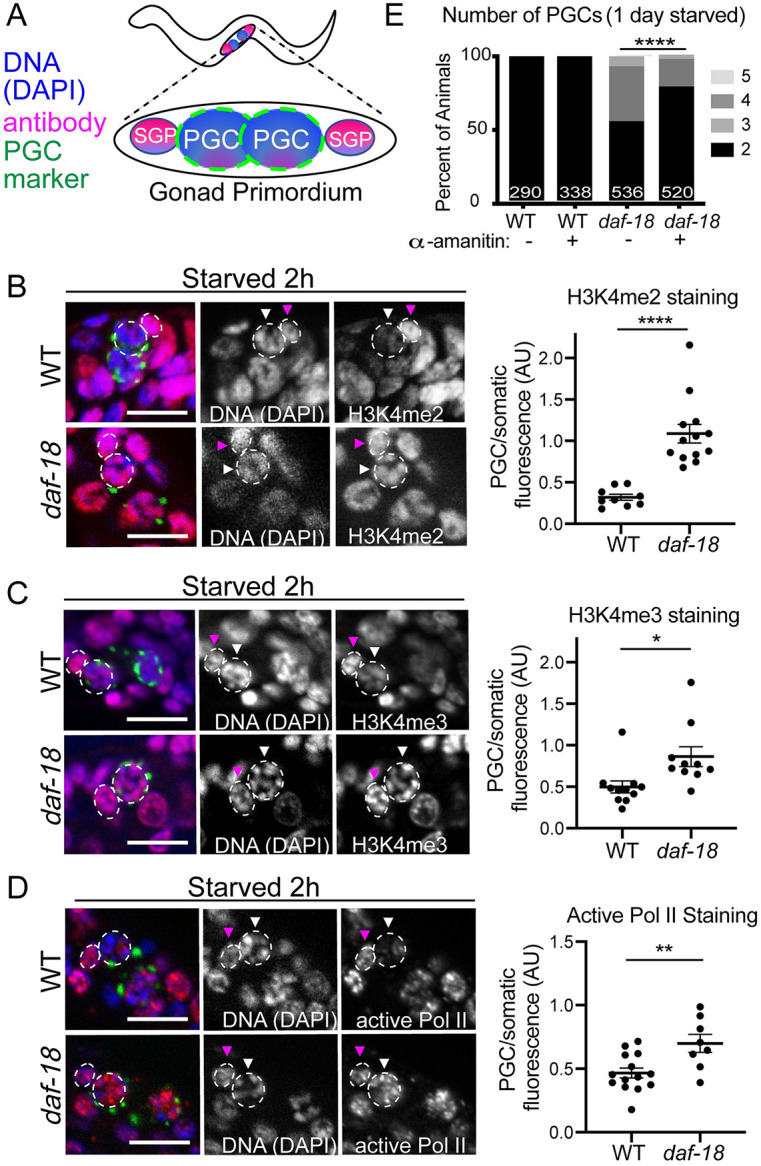
Marks of active transcription are inappropriately elevated in the PGCs of starved *daf-18* mutant L1 larvae. (A) Schematic of gonad primordium, containing 2 PGCs, each with 1 neighboring somatic gonad precursor (SGP), and the stains/marker seen in each tricolor image (left panels of B-D). PGC marker is *glh-1*::*GFP*. (B) Antibodies CMA303 (H3K4me2), (C) ab8580 (H3K4me3), and (D) H5 (Pol II P-ser2) were used to stain whole starved L1s starved up to 2 hours from hatching. (B-D) To illustrate PGC versus somatic fluorescence intensity, images in B and C show Z-projections of image stacks taken through the PGCs, while D shows single slices (Apotome). Tricolor images (left panels show DNA (DAPI/blue), immunofluorescence (magenta), and a PGC marker (encoded perinuclear *glh-1*::*GFP*). Dashed circle indicates one PGC in each image, and dashed smaller ellipse indicates its nearest somatic cell (likely its SGP). White arrowheads point to PGCs, and pink arrowheads point to neighboring somatic cells. Mean staining fluorescence was quantified for each PGC nucleus and its nearest somatic cell nucleus (from single image slices), and is displayed in the graph as a ratio. Each dot in the graph is an average value per worm (2 PGC/SGP values averaged). Statistical significance determined by two-tailed T-test. *p<0.05, **p<0.01, ****p<0.0001. (E) Transcription by Pol II was inhibited with alpha-amanitin (10μg/mL) and PGC numbers were assessed after 1 day (~24h) of starvation. Statistical significance determined by two-sided Fisher’s exact test. ****p<0.0001. All scale bars represent 10μm.

To determine whether these molecular marks are elevated in PGCs of *daf-18* mutants in the absence of food, we examined levels of antibodies detecting H3K4me2, H3K4me3, and active RNA Pol II in starved *daf-18(+)* and *daf-18(0)* relative to somatic cells. We found that while marks of H3K4me2 and H3K4me3 were detectable in the PGCs of starved wild-type worms, their levels were significantly lower relative to the surrounding somatic cells (Fig 3). In contrast, PGCs of starved *daf-18* mutants shortly after hatching and prior to cell division, displayed elevated levels of H3K4me2 and H3K4me3, comparable to the surrounding somatic cells (Fig 3B and 3C and S4 Fig). We also observed slightly elevated H3K4me2 in the PGCs of *daf-18* mutant 3-fold embryos, the stage just before hatching (S5A Fig). These observations suggest that the chromatin in *daf-18* mutant PGCs may be permissive for transcription during L1 starvation.

To assess whether transcription is likely occurring in PGCs of starved *daf-18* mutant L1 larvae, we examined the levels of P-Ser2. We detected low levels in PGCs of starved wild-type worms compared to surrounding somatic cells. Similar to what we observed with active chromatin marks, P-Ser2 is markedly elevated in PGCs in starved *daf-18* mutant L1 larvae, reaching levels roughly equivalent to nearby somatic cells (Fig 3D).

We wondered whether the *daf-18(+)* transgenes that promote PGC quiescence in *daf-18* mutants (*germline*::*daf-18(+)*, *daf-18 genomic(+)*, *intestine*::*daf-18(+)*, see Fig 2) might also result in reduced levels of active chromatin marks in the PGCs of *daf-18* mutants. Using the same immunohistochemistry strategy described above, with antibodies detecting H3K4me2 and H3K4me3, we measured relative levels of fluorescence in the PGCs and somatic cells of individual L1 worms. We found that *daf-18* mutants carrying germline-driven *daf-18(+)* displayed levels of H3K4me2/3 in the PGCs that were about half that of the nearest somatic cell levels (S6 Fig) resembling the wild type (Fig 3). By contrast, *daf-18* mutants expressing genomic *daf-18(+)* or intestinal *daf-18(+)* had significantly higher levels of H3K4me2/3 with respect to somatic cells, resembling the *daf-18* mutant (S6 Fig). These results suggest that germline DAF-18 restricts active germline chromatin while somatic DAF-18 appears to have little to no effect on germline H3K4me2/3 levels, despite significantly influencing PGC divisions.

### Inhibiting transcription suppresses PGC divisions in starved *daf-18* mutants

Since we observed that PGCs in starved *daf-18* mutants display inappropriately high levels of marks of transcriptional activation around the time of hatching and therefore well before the time that PGCs would divide, we hypothesized that inhibiting transcription would block inappropriate PGC divisions. To test this hypothesis, we inhibited Pol II using α-amanitin [53,54]. We allowed *daf-18* mutant and wild-type embryos to hatch in buffer with no food, with or without α-amanitin. This treatment significantly reduced the proportion of starved *daf-18* mutant animals with ≥3 PGCs after one day of L1 starvation (Fig 3E), without affecting survival of the animals (S7 Fig). Taken together with the elevated marks of active transcription, these results suggest that activation of PGC transcription precedes inappropriate PGC divisions in starved *daf-18* mutants and may be required for such divisions.

### Levels of zygotic VBH-1 and GLH-1 are inappropriately elevated in PGCs of starved daf-18 mutants prior to cell division

**Fig 4:**
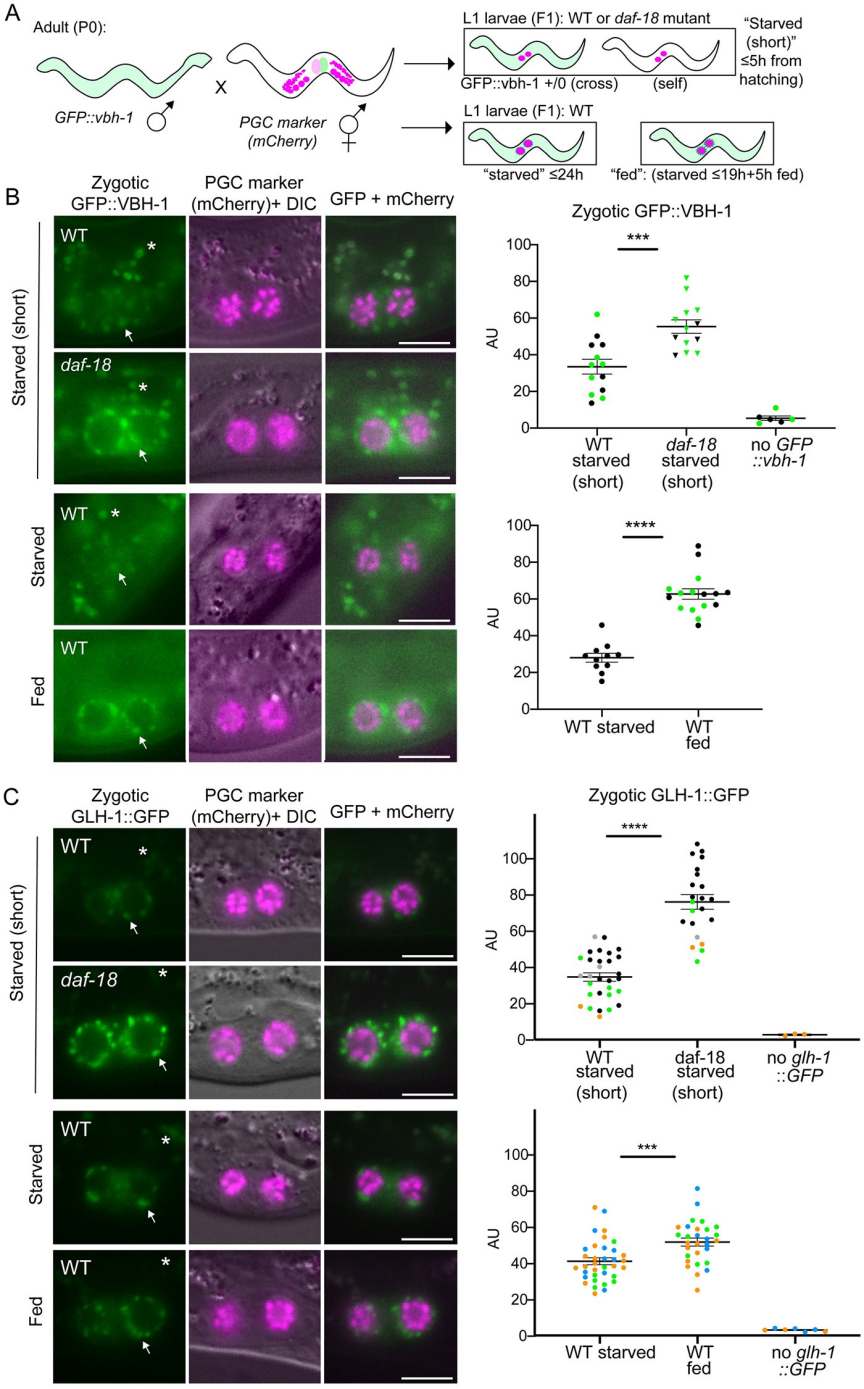
Levels of zygotic VBH-1 and GLH-1 are inappropriately elevated in PGCs of starved *daf-18* mutants. (A) Schematic of strategy to observe strictly zygotic expression of germline genes *vbh-1* and *glh-1*, tagged with GFP. Single-copy GFP inserted into endogenous *vbh-1* or *glh-1* loci was crossed from the male into *PGC*::*mCherry* hermaphrodites. Crosses were performed in either the wild type (WT) or *daf-18(ok480)* mutant background. “Starved (short)” L1 progeny were imaged up to 5 hours after hatching (with no food). “Starved” progeny were collected and imaged up to 24 hours after clean embryo preparation, while “fed” L1 progeny were similarly imaged at 24 hours (≤24 hours), but the last 5 hours prior to imaging, they were on food (up to 19 hours starved + 5 hours fed; (see Methods). (B) Representative images taken within 1 hour of hatching. Arrows point to zygotic perinuclear GFP::VBH-1 in PGCs. Exposure time is longer in B than in C, so gut granules (green auto-fluorescent dots, marked with asterisks and visible in DIC) are more prominent. Graphs show the results of two experiments pooled together (dots color-coded per experiment). Similar results for WT vs. *daf-18* starved were observed in an additional experiment on L1 larvae starved up to 24h. (C) Representative images taken up to 5 hours after hatching. Graphs show the results of 5 experiments (dots color-coded per experiment). Arrows point to zygotic perinuclear GFP::VBH-1 in PGCs. Gut granules (green auto-fluorescent dots) are marked with asterisks and visible in DIC. (B and C) Images show epifluorescence. Mean perinuclear GFP fluorescence intensity (AU = Arbitrary Units) values are plotted to the right of each set of images. Each dot represents one animal (either average of 2 PGC values, or one PGC per animal). Non-cross progeny were identified by the lack of somatic GFP (“no GFP::vbh-1”) or lack of any GFP P-granules (“no GLH-1::GFP”; this transgene is much brighter and is clearly visible in starved WT PGCs). Mean +/- SEM. Statistical significance determined by two-tailed T-test. ***p<0.001 ****p<0.0001. All scale bars represent 10μm.

It was previously shown by mRNA-FISH that the transcription of genes encoding five different P granule-associated proteins is induced in PGCs upon L1 feeding [55]. To observe germline expression in live worms, we tagged one of them, VBH-1 (Vasa and belle-like RNA helicase) with GFP using CRISPR/Cas9. To assess zygotic expression of the GFP::VBH-1 protein fusion, we crossed it in from the male, creating (M-Z+) F1 progeny carrying one paternal copy of GFP::VBH-1. Since these F1 progeny come from non-GFP mothers, any green fluorescence is the result of zygotic expression (Fig 4A). In the wild-type genetic background, zygotic perinuclear GFP::VBH-1 was only barely detectable in starved L1 PGCs. In contrast, PGCs in starved *daf-18* mutants displayed bright zygotic GFP::VBH-1. The intensity of this perinuclear fluorescence is equivalent to that of fed wild-type animals, and is observed within one hour of hatching, several hours before the PGCs begin dividing. In wild-type animals, after 4–5 hours of feeding, strong perinuclear expression was observed in the PGCs (Fig 4B).

To determine when *daf-18* mutants begin to inappropriately express zygotic GFP::VBH-1, we analyzed embryos. We observed that zygotic GFP::VBH-1 was faintly visible in many somatic cells, in both wild-type and *daf-1*8 mutant embryos. However, it was not readily detected in the PGCs above somatic levels. The exceptions were a handful of late-stage (3-fold) embryos (3 of 10 embryos) (S5 Fig). Thus it is possible that *daf-18* mutant embryos begin to express GFP::VBH-1 just before hatching.

Since VBH-1 is broadly expressed at a low level in most somatic tissues in addition to its high expression in the germ line [41,45], we wondered whether *daf-18* mutants also zygotically express inappropriately high levels of GFP::VBH-1 in the soma. We observed faint, diffuse cytoplasmic expression of zygotic GFP::VBH-1 in somatic cells of the wild-type L1, particularly in head neurons. However, we did not observe significant differences in this head expression between starved *daf-18* mutants and the wild type (S8A Fig), suggesting that zygotic somatic VBH-1 expression is not regulated by *daf-18*.

To determine whether the inappropriate germline zygotic expression seen in starved *daf-18* mutant PGCs was specific to VBH-1, we investigated another germline gene reporter: GLH-1::GFP (Germ Line Helicase), a component of germline-specific P granules [56]. We used an available GLH-1::GFP CRISPR allele [57], and crossed it in from the male to examine zygotic expression. Like GFP::VBH-1, we found that in PGCs of starved *daf-18* mutant animals, the level of zygotic GLH-1::GFP expression was significantly elevated compared to the wild type within a few hours of hatching (Fig 4C).

In addition, we observed several differences between the germline zygotic activation of VBH-1 and GLH-1. GFP::VBH-1 is barely detectable in PGCs prior to feeding in the wild type and feeding strongly elevated expression relative to the starved condition (Fig 4). By contrast, the germline zygotic expression of GLH-1::GFP was relatively high in starved wild-type L1 worms. This is not completely surprising, since a previous report suggests that *glh-1* is transcribed zygotically in the embryo [58]. Nevertheless, zygotic GLH-1 levels are markedly elevated upon feeding. We also found that while the levels of expression of VBH-1 in PGCs of starved *daf-18* mutants are comparable to fed wild-type larvae, GLH-1 is expressed at a higher level in starved *daf-18* mutants than in the fed wild type. This observation suggests that *daf-18(+)* may contribute to a food-independent effect on germline zygotic gene expression.

We reasoned that if *daf-18* mutants elevate transcription of all germline genes, they should also express inappropriately high levels of our single copy *PGC*::*mCherry* marker (Fig 1A) that is driven by the *mex-5* promoter. In contrast to *vbh-1* and *glh-1*, zygotic *mex-5P*::*mCherry* was expressed at a low and similar level in the PGCs of both starved and fed L1 larvae, as well as in starved *daf-18* mutants (S8 Fig). This suggests that not all germline genes are up-regulated by the loss of *daf-18*.

In summary, our results together with those of Wong et al. (2018) suggest that DAF-18 represses germline zygotic activation of a subset of genes in the absence of food.

### Germline identity appears unaffected by *daf-18*

One possible explanation for the elevated zygotic gene expression in PGCs is that loss of *daf-18* may compromise germline identity such that the PGCs express genes zygotically on a schedule similar to the soma. If so, zygotic somatic gene expression might occur in PGCs of *daf-18* mutants. To test this hypothesis, we investigated zygotic expression of *unc-119P*::*GFP*, a neuronal gene reporter previously shown to be expressed inappropriately in the germ line under conditions where germ cell identity is compromised [59–62]. We found that zygotic *unc-119P*::*GFP* is not detectable in PGCs, neither in the starved or fed wild type, nor the starved *daf-18* mutant (S8 Fig). On the other hand, starved wild-type animals show zygotic *unc-119P*::*GFP* expression in neurons, and the fluorescence intensity of this expression was slightly elevated in *daf-18* mutants. In addition, we did not observe any *unc-119P*::*GFP* expression in the adult germ line of continuously fed wild-type or *daf-18* mutant worms as adults, and in no case did we see the germline-specific *glh-1* or *mex-5* reporters expressed outside of the germ line in *daf-18* mutants. Therefore, within the limits of these assays, we found no evidence for altered germline-soma identity in *daf-18* mutants.

### Transcript levels of a subset of germline genes are elevated in starved *daf-18* mutants

**Fig 5:**
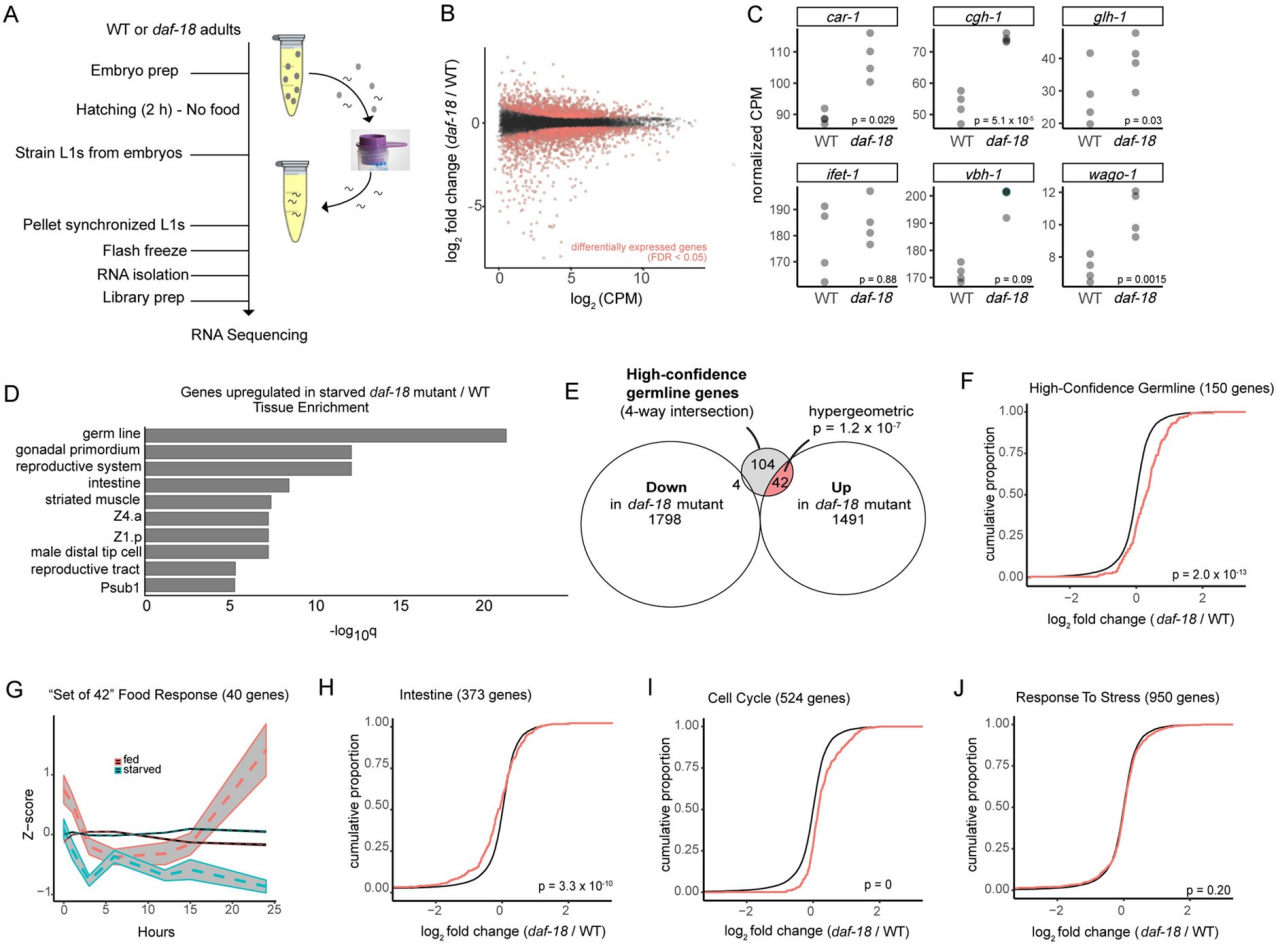
Transcript levels of a subset of germline genes are elevated in starved *daf-18* mutants. (A) Schematic of RNA-sequencing experiment. Starved L1s were isolated within 2 h of hatching, pelleted, frozen, and mRNA sequencing was performed. (B) Plot of log_2_ fold change (in GC1459 *daf-18(ok480)* / GC1171 wild type (WT) versus expression level of 12,592 transcripts detected. Red color dots represent transcripts expressed differentially (below cutoff of FDR 0.05). (C) Transcript levels of genes previously shown to be induced zygotically by feeding [55] or by *daf-18* mutation (this study). Uncorrected p-values are displayed since comparisons were hypothesis-driven. (D) Tissue enrichment analysis of genes upregulated in *daf-18* mutant/WT using WormBase web tool. Germ line is the most significantly represented tissue in *daf-18* mutant-upregulated genes. (E) Venn diagram of transcripts significantly upregulated or downregulated in *daf-18* mutants (FDR<0.05) and overlap with the set of “high-confidence germline genes,” or transcripts found in all of 4 published germline sets [41,45,58,65]. Hypergeometric p-value is displayed for *daf-18* up-regulated genes overlap with high-confidence germline genes (while p = 1 for overlap of *daf-18* downregulated genes with high-confidence germline genes). (F-I) Cumulative distribution function (CDF) plots of transcript sets of interest (red line) versus the background set of all 12,592 transcripts detected (black line). Shift to the right indicates overall increased expression of gene set. p-values determined by Kolmogorov-Smirnov (KS) test are shown. Numbers of transcripts from published gene sets detected in our experiment are shown in graph titles (parentheses). (G) Fed versus starved L1 expression (data from Baugh et al., 2009) of our set of DAF-18-repressed high-confidence germline genes. Mean Z-score (and 95% confidence intervals) of these 40 genes (of 42 we identified in E also detected in this study) over time are plotted in grey, while the thin, black lines represent the Z-scores for the entire background set (11,107 genes from Baugh et al., 2009 that were also detected in this study). (H) Intestine gene set [45], data accessed by GExplore1.4 web tool) shows significant differential expression in *daf-18*, but many genes are downregulated. **I.** and **J.** show significant upregulation (Cell Cycle) and no significant change (Response to Stress) of gene sets of interest.

We reasoned that if *daf-18* mutant PGCs robustly activate germline zygotic gene expression despite or even prior to starvation, more pervasive effects on germline transcript levels should be detectable. To test this prediction, we used a combined mRNA-sequencing and bioinformatics approach to compare germline and somatic gene expression. We compared transcript levels from whole, starved *daf-18* mutant and wild-type L1 larvae within two hours of hatching, in four independent biological replicates (Fig 5A). We then used available annotation of tissue-specific expression and gene function to analyze the results. Principal Component Analysis and pairwise correlations demonstrated reproducibility of replicates and a relatively large effect of *daf-18* on gene expression (S9 Fig), with the first principal component clearly separating the two genotypes and explaining over half of the variance. 3,335 genes were significantly differentially expressed (false discovery rate (FDR) < 0.05) (Fig 5B); 1,802 genes were down-regulated and 1,533 up-regulated in *daf-18* mutants relative to the wild type (S1 Data).

To focus our comparative analysis on germline genes, we performed several independent analyses. First, we examined the five genes that Wong et al., 2018 had shown by mRNA FISH to be up-regulated upon feeding (*vbh-1*, *car-1*, *cgh-1*, *wago-1*, *ifet-1*), plus *glh-1* that, like *vbh-1*, had elevated zygotic expression in starved *daf-18* mutants based on live GFP protein fusion reporters (Fig 4). *car-1*, *cgh-1*, *wago-1*, and *glh-1* were significantly up-regulated in starved *daf-18* mutants compared to the wild type (Fig 5C). *vbh-1* and *ifet-1* were also elevated, but not to the level of statistical significance. This may be related to their somewhat broader tissue expression in the soma compared to the other four genes [45].

Second, we performed an unbiased analysis to determine which tissues were most highly represented by the list of genes upregulated in *daf-18* mutants. Using the Enrichment Analysis web tool provided by WormBase [63,64], https://wormbase.org/tools/enrichment/tea/tea.cgi), we found that the germ line clearly showed the most significant enrichment in the genes for which expression went up in *daf-18* mutants (Fig 5D).

Third, to identify a set of high-confidence germline-enriched genes, we took advantage of four independent lists of genes previously reported to be enriched or expressed in the germ line [41,45,58,65] (see Methods for details). Our experiment detected transcripts of a large percentage of the genes in these sets (S10 Fig and S1 Data for all genes detected and statistical analyses). We found that these four independent analyses identified 167 germline genes in common, 150 of which were detected in our experiment. We refer to these as the “high-confidence germline gene” set. Notably, of the 46 genes in this set that were differentially expressed, 42 were present at significantly higher levels in the *daf-18* mutant (Fig 5E). In addition, a cumulative distribution function (CDF) plot of all genes in the high-confidence germline set also revealed a significant shift toward higher expression for the set of high-confidence germline genes in *daf-18* mutants (Fig 5F). Each independent germline-gene set [41,45,58,65] also showed a significant shift toward higher expression in *daf-18* mutants (S10 Fig).

To investigate the relationship between *daf-18* and feeding-dependent transcription, we asked whether the 42 high-confidence germline genes identified as having elevated transcript levels in *daf-18* mutants during starvation (“set of 42”; Fig 5E) are also food-responsive genes. We analyzed levels of expression of these 42 genes in a published data set: a transcriptome time course of wild-type L1s for 24 hours after hatching, in the presence or absence of food [66]. Of the 11,107 genes from our current study that were also detected in the Baugh 2009 data set, 40 of the set of 42 were included. The transcript abundance of these 40 genes, comparing starved and fed (in WT animals), begins to diverge around 6 hours, and then more markedly diverges after about 15 hours of feeding (Fig 5G shows the average Z-score of the 40 genes plotted against the background set, and S11 Fig shows the individual genes’ Z-scores). Notably, 37 of these 40 genes were differentially expressed over time with higher expression in fed than starved wild-type larvae, compared to 112 of the 142 high-confidence germline genes detected in this dataset, and the GO term enrichments for the 37 and 112 gene sets were very similar. Although the increase in transcript abundance of these germline genes in fed worms seems surprisingly late given that PGC divisions begin at ~4h of feeding, it is possible that limits of transcript detection and time to accumulate signal, especially with less precise staging and microarray analysis, may account for the delayed increase. Nevertheless, these results suggest that many of the same germline gene transcripts that are significantly more abundant within two hours of hatching in starved *daf-18* mutants (relative to starved wild type) are also elevated in wild-type animals upon feeding.

To determine whether genes expressed in specific somatic tissues were similarly affected we used the GExplore1.4 web tool [45,67] http://genome.sfu.ca/gexplore/gexplore_search_all.html) to select genes expressed in the intestine, ciliated neurons, touch receptor neurons, non-seam hypodermis, seam cells, body wall muscle and pharyngeal gland (all are reported to express *daf-18* except for touch receptor neurons according to GExplore data, and possibly pharyngeal gland cells [41]). In each case, RNA sequencing detected the majority of the genes in these lists (S1 Data). We found significant differential gene expression in *daf-18* versus the wild type for genes expressed in the intestine, ciliated neurons, touch receptor neurons, and non-seam hypodermis. However, as shown by the CDF plots, none of these gene sets showed the same overall shift toward elevated expression as was seen with the high-confidence germline gene sets (Fig 5H shows intestine; other tissues are shown in S12 Fig).

We then asked whether genes associated with relevant GO terms showed differential expression in *daf-18* mutants. We selected GO terms associated with the germ line (Reproductive Process, Germ Cell Development, P granule, Cytoplasmic Stress Granule, Gene Silencing by RNA), as well as terms relevant to *daf-18* and/or PTEN function (Cell Cycle, Response to Stress, DNA Damage, Translation). Finally, we looked at genes annotated with both Translation and Reproductive Process GO terms to assess translation within the germ line. Based on our analysis of CDF plots, we found that genes in each of these GO term categories were up-regulated in starved *daf-18* mutants, with the exception of the “cytoplasmic stress granule” and “response to stress” classes. We were not surprised to see cell cycle genes upregulated (Fig 5I), given that the cells that divide in fed L1 larvae, including germ cells and some somatic cells, proliferate inappropriately in starved *daf-18* L1 larvae [5,31,34]. The “cytoplasmic stress granule” class contained only 15 genes, limiting statistical power, so interpretation of this finding is difficult (S12 Fig). However, our failure to detect differential gene expression in the “response to stress” set of 950 genes suggests that the loss of *daf-18* does not appreciably alter the animal’s global stress response at the transcriptional level (Fig 5J). Genes annotated with the other seven GO terms examined were significantly differentially expressed (upregulated overall) (S12 Fig).

We also performed an unbiased analysis of gene ontology (GO) terms, using WormBase’s Enrichment Analysis Tool. We found that among the genes upregulated in starved *daf-18* mutants, some of the most significantly enriched GO terms were related to metabolism and cell division (S12 Fig).

Taken together, our results from this RNA-seq experiment, as well as zygotic gene-reporter expression experiments, indicate that DAF-18 plays a major role in suppressing expression of germline genes during starvation. This effect appears to be restricted to the germ line, though it is possible that some germline genes are upregulated in the soma.

### Regulation of germline zygotic gene activation by DAF-18 is partially independent of TOR

**Fig 6:**
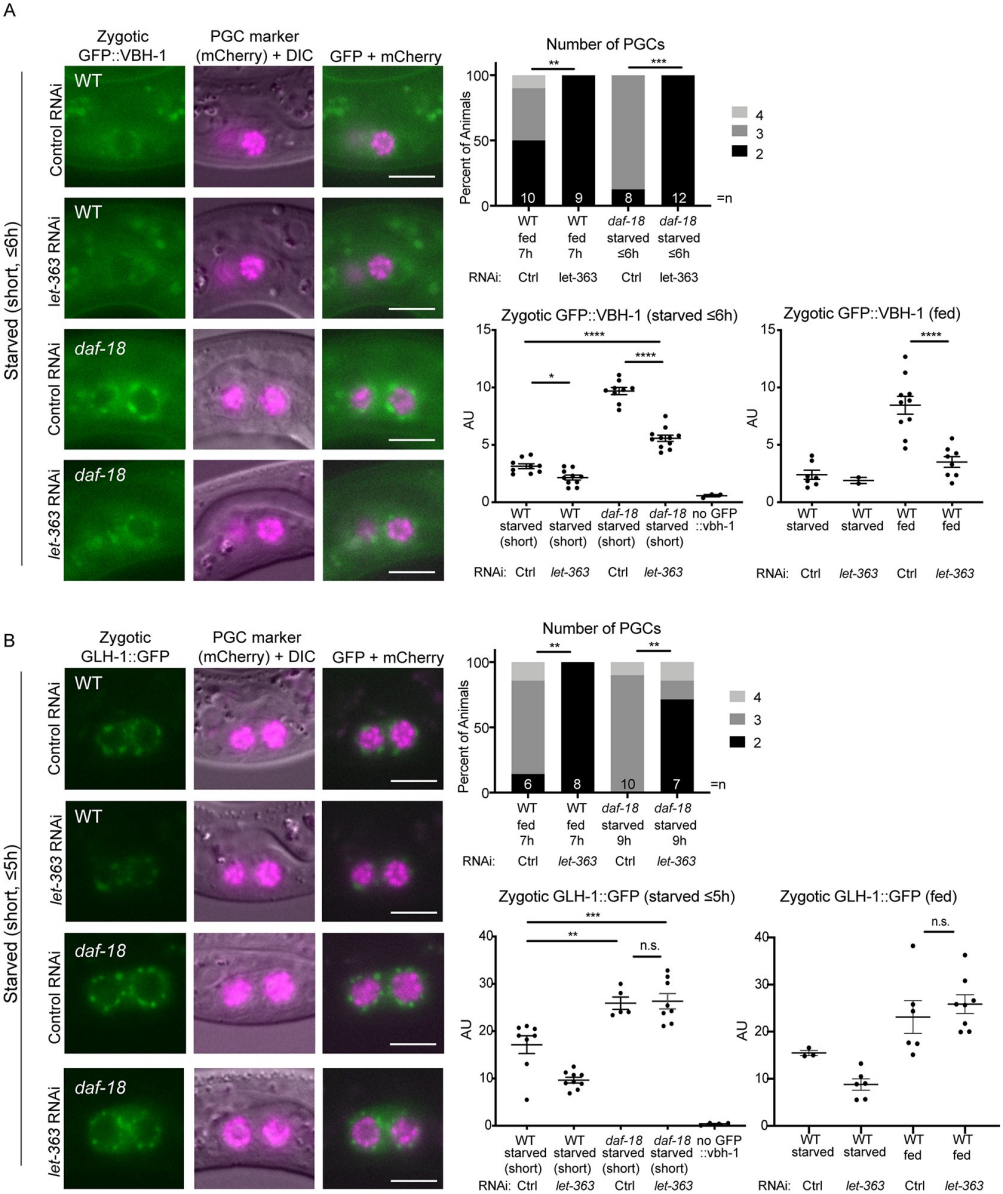
Regulation of germline zygotic gene expression by DAF-18 is partially independent of TOR. (A-B) Crosses were performed as in Fig 4, but on RNAi bacteria (RNAi by feeding), and L1 progeny analyzed, either starved (images and graph) or fed (graph only). RNAi bacteria carried dsRNA to *let-363*/TOR or Control (“Ctrl”; empty vector L4440). Images show epifluorescence. Plotted to the right of each set of images are 1) plots of PGC numbers to illustrate efficacy of *let-363*/TOR RNAi in that experiment, and 2) graphs of mean perinuclear GFP fluorescence intensity (AU) values for starved or 3) fed L1 progeny. For PGC number graphs, statistical significance determined by Fisher’s exact test (two-sided). **p<0.01, ***p<0.001. For zygotic GFP images, exposure time is longer for GFP::VBH-1 than GLH-1::GFP. For GFP quantification graphs, each dot represents one animal (either average of 2 PGC values, or one PGC per animal). Non-cross progeny were identified by the lack of somatic GFP (“no GFP::vbh-1”) or lack of any GFP P-granules (“no GLH-1::GFP”; this transgene is much brighter and is clearly visible in starved WT PGCs). Mean +/- SEM. Statistical significance determined by one-way ANOVA (Tukey’s multiple comparisons test).*p<0.05, **p<0.01, ***p<0.001, ****p<0.0001. All scale bars represent 10μm. For these experiments, the exact timing of image analysis is as follows (see Methods): “Starved (short)” L1 progeny were imaged up to 6 hours after hatching. “Starved” and “fed” L1 progeny were imaged up to 22 hours after clean embryo preparation; the last 7 hours of which for “fed” were on food. For (A), two additional replicates showed similar results (slight suppression of elevated GFP::VBH-1 in *daf-18* mutants, by TOR RNAi). To assess *let-363*/TOR RNAi efficacy, PGC numbers were counted in L1s starved up to 6 hours. For (B), one additional replicate showed similar results (no suppression of elevated GLH-1::GFP in *daf-18* mutants, by TOR RNAi), and PGC numbers were counted in L1s starved up to 9 h.

Previous results showed that depleting *let-363*/TOR by RNAi significantly suppresses the inappropriate cell division phenotype of *daf-18* mutants [32]. We wondered whether interfering with TOR by RNAi would similarly suppress the inappropriate zygotic germline gene expression in PGCs of starved *daf-18* mutants. To test this hypothesis, we depleted *let-363*/TOR by RNAi and assayed both PGC division and zygotic expression. We initiated RNAi feeding in the L4 stage of hermaphrodites, crossed the GFP-tagged VBH-1 or GLH-1 fusion from the male, and scored the starved L1 progeny for the number of PGCs and the expression level of the tagged protein. As an additional control for the efficacy of RNAi, we also examined progeny of these matings that had been continuously fed on RNAi or control bacteria in parallel. While the progeny exposed to control bacteria became fertile adults, we observed completely penetrant larval arrest on *let-363* RNAi, mimicking the null mutant phenotype [68] and thereby confirming good efficacy of the RNAi. We also confirmed that *let-363* RNAi suppresses PGC divisions in starved *daf-18* mutant L1 larvae (Fig 6), as was shown previously [32]. We also noted that PGC divisions did not occur in fed wild-type L1 larvae upon *let-363* RNAi within the time frame of our scoring.

*let-363* RNAi affected zygotic VBH-1 expression in two ways. First, we found that VBH-1 levels were reduced in the PGCs in fed wild-type larvae. Second, the elevation of VBH-1 in *daf-18* mutants relative to the starved wild type was only partially suppressed by *let-363* RNAi (Fig 6A).

We used the same RNAi experimental design to test whether inappropriately elevated GLH-1 zygotic expression in starved *daf-18* mutants is regulated by *let-363/*TOR. We found that, despite strong suppression of PGC divisions in starved *daf-18* mutants, upon *let-363*/TOR RNAi, neither fed WT nor starved *daf-18* mutant animals showed a reduction of zygotic GLH-1 levels (Fig 6B).

**Fig 7:**
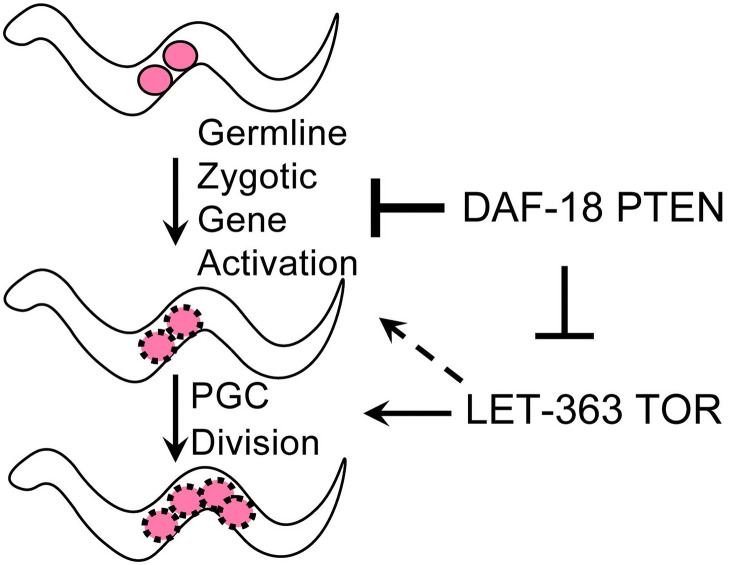
Model for DAF-18-mediated regulation of arrested PGCs in L1 starvation conditions. In the absence of food, DAF-18 represses germline zygotic gene activation, partially independent of its canonical negative regulation of PI3K-TOR that regulates PGC division. See text for details.

Together, our results are consistent with a model (Fig 7) in which DAF-18 impedes germline zygotic gene activation independently of its negative regulation of TOR via opposition of PI3 kinase activity, though both activities ultimately influence PGC division.

## Discussion

We investigated several aspects of DAF-18 PTEN function in maintaining PGC quiescence in the absence of food. First, we found that the control of PGC division appears to be remarkably distributed, both between different tissues and also between maternal and zygotic *daf-18* provision. Most striking, we observed nearly complete rescue from a transgene expressing *daf-18(+)* in the germ line and partial suppression from several somatic tissues including the somatic gonad and intestine. These findings distinguish the anatomical requirements for *daf-18* in PGC quiescence from its later role in restricting germ cell proliferation during dauer where *daf-18* acts solely in the somatic gonad to regulate germline proliferation [28]. Though germ cells arrest in G2 in both cases, this difference underscores differences in regulation of germ cell quiescence in L1 PGCs and later germ cells that have undergone proliferation prior to dauer entry. We also found that either maternal or zygotic *daf-18(+)* appears sufficient to maintain PGC quiescence. While perplexing, one possibility is that this level of redundancy may have evolved as a failsafe to ensure PGC quiescence in the absence of food.

Second, our results point to a possible role for DAF-18 in repressing gene expression in PGCs. Furthermore, they suggest that DAF-18-mediated repression of germline gene expression and PGC cell cycle progression in response to food are not inextricably linked. That is, the role of DAF-18 in repressing gene expression in the germ line is partially independent of its role opposing TOR signaling.

Third, we found that, within the limits of our assay of *unc-119* expression, which is sensitive to germline-to-soma transformation, there is no evidence for *daf-18* activity in regulating the germline-soma identity decision. Nevertheless, the proliferated germ cells in *daf-18* mutants are likely abnormal.

We propose a model in which DAF-18, through as-yet unknown molecular mechanisms, restrains zygotic gene activation in the germ line to interfere with exit from quiescence. Our model rests on the following findings. First, even when worms hatch on food, PGCs of *daf-18* mutant worms initiate divisions earlier than in the wild type, suggesting that they are primed to divide. Second, several germline genes are more highly expressed in PGCs both upon feeding ([55]; this study) and in starved *daf-18* mutants (this study). Third, we found that several global indicators of active transcription are elevated inappropriately in PGCs of newly hatched *daf-18* mutants, and active transcription is required for full penetrance of the inappropriate PGC divisions in starved *daf-18* mutants, based on chemical inhibition. Fourth, some germline gene transcripts, as a group, appear to be present in greater abundance in starved *daf-18* mutant hatchlings relative to the starved wild type. That said, while only the PGC expression (and not somatic expression) of VBH-1 appeared to be elevated by loss of *daf-18*, our RNA-sequencing results show that transcript levels of several other germline genes were not elevated in *daf-18* relative to the wild type (e.g., *nos-1*, *nos-2* and *mex-5*; the latter is consistent with our zygotic expression analysis of a *mex-5* driven promoter, S8 Fig). We also note that some somatic transcripts are elevated in starved *daf-18* mutants, such as *unc-119* (consistent with zygotic transgene expression analysis S8 Fig). Nonetheless, in general, over 90% of differentially regulated genes within the high-confidence set of germline genes are up-regulated rather than down-regulated in *daf-18* mutants. We note that our RNA-seq analysis was performed on L1 larvae just after hatching, and therefore our observations are likely unrelated to differences in germline transcript stability that are observed upon longer-term starvation [69]. Last, levels of germline zygotic gene expression (VBH-1 and GLH-1) in PGCs of starved *daf-18* mutants are elevated even when *let-363* TOR is depleted.

Our model raises many questions for future studies. For example, do the cell cycle and transcriptional regulatory activities of *daf-18* on L1 germ cell quiescence act from the same tissues? Do they result from different subcellular localization and/or biochemical activities of DAF-18 PTEN? For example, in neuroblast divisions, DAF-18 acts via PI3K but upstream of MAPK [34], whereas in oocyte maturation, DAF-18 acts downstream of the VAB-1 Ephrin receptor and upstream of MAPK, independent of AKT and lipid-phosphatase activity [38]. It is possible that the different biochemical activities of DAF-18 correlate with their anatomical requirements. For example, the lipid phosphatase role of DAF-18 PTEN could correlate with post-transcriptional cell cycle control via regulation of PI3K signaling and TOR dependence while a germ-cell autonomous function of DAF-18 PTEN (perhaps as a protein phosphatase) could responsible for the transcriptional repression in the germ line. Likewise, the maternal or zygotic requirements may be functionally separable. A related unresolved issue is why maternal expression of the broadly-expressed genomic transgene rescues PGC divisions in progeny that have lost the transgene, but this transgene does not appear to be expressed in the germ line, nor does it appear to influence H3K4 methylation. The anatomical requirements for this rescue are unclear, as is the possible role of transgene-mediated overexpression. One possibility is that the genomic transgene is expressed in the germ line at a level below detection, and that this level of germline expression is insufficient to measurably affect germline histone methylation. Additional studies are required to resolve the mechanisms of DAF-18 function for its effects on PGC division and germline transcription.

Several other scenarios permit inappropriate PGC division. In the embryo, depletion of *nos-1* and *nos-2* can cause PGCs to divide prematurely [70]. However, these divisions likely reflect defects in fate specification [65] rather than alterations in levels of germline transcripts. In addition, depletion of the basement membrane component laminin allows PGCs to divide [71]. However, this phenotype does not likely relate to DAF-18 since, unlike PGC divisions in the *daf-18* mutant [5], loss of *akt-1* does not suppress PGC divisions that occur upon depletion of laminin. Rather, inappropriate PGC divisions are dependent on GLP-1 Notch signaling [71]. We examined our RNA-seq dataset and found that both *sygl-1* and *lst-1* (the relevant outputs of GLP-1 signaling) are indeed detected but their levels are not significantly different in starved *daf-18* mutants versus wild type.

Like loss of *daf-18*, loss of AMPK (*aak-1* and *aak-2* double mutants, referred to here as *aak*) causes PGC divisions and marks of active transcription in L1 larvae that hatch in the absence of food [32,72]. However, these pathways act at least partially independently with respect to their effects on PGC divisions, post-starvation fertility and post-starvation survival. For each of these post-starvation phenotypes, the loss of both *aak* and *daf-18* together causes a more severe defect than the loss of either pathway alone [32,72]. Moreover, regulation of PGC divisions and their relationship to post-starvation fertility differ. Whereas loss of *age-1* PI3-kinase suppresses both the inappropriate PGC divisions and post-starvation sterility of *daf-18* mutants, it does not restore fertility to the aak double mutant. Additionally, although loss of *raga-1* prevents PGC division in both *daf-18* and *aak* mutants, it restores post-starvation fertility to *daf-18* but not to aak [32,72]. Finally, *aak* mutants display a transgenerational brood size defect that is not observed after starvation in *daf-18* mutants. In the *aak* mutants, this latter phenotype has been linked to elevated levels of H3K4me3, the deleterious effects of which in the germ line are dependent on the COMPASS complex [72], which permits the expression of germline genes. While there are clearly differences in the roles of DAF-18 PTEN and AAK AMPK in the PGCs, it will be of interest to compare how each acts on chromatin and the consequences on germline gene transcription and expression.

How does DAF-18 interfere with germline zygotic gene activation in PGCs? And what releases this block? The trigger(s) for germline zygotic gene activation are mysterious. In worms, flies, fish, frogs and mammals, germline zygotic gene activation occurs after zygotic genome activation, and, similar to zygotic activation in the soma, it likely requires both degradation of maternal mRNAs and activation of a zygotic program of transcription. Degradation of maternal mRNAs is an important step in securing germ line identity in *C*. *elegans* [65].

While future work is required, several general possibilities can be imagined, none of which are mutually exclusive, as outlined below. Regardless of whether DAF-18 acts biochemically as a lipid phosphatase or protein phosphatase, or otherwise, a downstream effect of DAF-18 could alter one or more processes that promote germline zygotic gene expression. One possibility is that DAF-18 changes the activity state or localization of one or more transcription factors. We noticed, for example, that according to the WormExp [73], 37 of the 46 high-confidence germline genes that were differentially expressed in *daf-18* mutants are also down in *ceh-23* mutants [74]. Another hypothesis that could account for the large number of activated germline genes is that DAF-18 may interact with chromatin, DNA, or components of transcription complexes, including RNA Pol II, to alter transcription at specific loci, as proposed for nuclear PTEN in mammalian cell culture [75]. DAF-18 may also have a more global effect on chromatin conformation, either via histone modifications or regulation of the degree of physical compaction of chromatin. This hypothesis is intriguing since the timing of chromatin decondensation correlates with germline zygotic gene activation in *C*. *elegans* PGCs in response to feeding [49,55]. There is also precedent in mammalian cell lines for PTEN involvement in chromatin condensation via interaction with linker histone H1 and stabilization of HP1alpha, both independent of its lipid phosphatase activity [76,77]. Condensed chromatin may be an evolutionarily conserved mechanism for securing the quiescent state, as it is also implicated in yeast and human cell lines [78]. Small RNAs and nuclear RNAi factors were recently shown to induce chromatin compaction in later stage germ cells [79,80]). We speculate that the same mechanisms used by DAF-18 to restrict germline zygotic gene expression in *C*. *elegans* could contribute to the competence of cells arrested in G2 to escape quiescence in the absence of PTEN in cancer.

## Methods

### Worm handling and strains

*C*. *elegans* strains were maintained on nematode growth medium (NGM) plates at 20°C and fed a diet of OP50 *E*. *coli* bacteria, unless noted otherwise [81]. Experiments were carried out at room temperature (~22°C).

#### Strains used in this study

Strains generated for this study are indicated below. All strains used and details on strain construction information are listed in S1 Table. **GC1459** *naSi2 II; unc-119(ed3) III*?*; daf-18(ok480) IV*, **GC1527** *glh-1(sam24[glh-1*::*gfp*::*3xFLAG]) I; daf-18(ok480) IV*, **GC1537** *vbh-1(na110[GFP*::*vbh-1]) I; naSi2 II*, **GC1576** *vbh-1(na110[GFP*::*vbh-1]) I; naSi2 II; daf-18(ok480) IV*, **GC1456** *naSi2 II; unc-119(ed3) III*?*; daf-18(ok480) IV; naEx261 (daf-18P*::*daf-18*::*daf-18 3’UTR*::*SL2*::*GFP*::*H2B*::*unc-54 3’UTR)*, **GC1460** *naSi2 II; unc-119(ed3) III*?*; daf-18(ok480) IV; naEx264 (rab-3P*::*daf-18*::*daf-18 3’UTR*::*SL2*::*GFP*::*H2B*::*unc-54 3’UTR)*, **GC1464** *naSi2 II; unc-119(ed3) III*?*; daf-18(ok480) IV; naEx268 (nlp-40P*::*daf-18*::*daf-18 3’UTR*::*SL2*::*GFP*::*H2B*::*unc-54 3’UTR)*, **GC1465** *naSi2 II; unc-119(ed3) III*?*; daf-18(ok480) IV; naEx269 (dpy-7P*::*daf-18*::*daf-18 3’UTR*::*SL2*::*GFP*::*H2B*::*unc-54 3’UTR)*, **GC1485** *naSi2 II; unc-119(ed3) III*?*; daf-18(ok480) IV; naEx275 (elt-2P*::*daf-18*::*daf-18 3’UTR*::*SL2*::*GFP*::*H2B*::*unc-54 3’UTR)*, **GC1484** *naSi2 II; unc-119(ed3) III*?*; daf-18(ok480) IV; naEx274 (ehn-3P*::*daf-18*::*daf-18 3’UTR*::*SL2*::*GFP*::*H2B*::*unc-54 3’UTR)*, **GC1479** *naSi22 (mex-5P*::*daf-18*::*nos-2 3’UTR*::*SL2*::*GFPo*::*PH*::*tbb-2 3’UTR + unc-119(+)) II; unc-119(ed3) III*?*; daf-18(ok480) IV*, **GC1483** *glh-1(sam24[glh-1*::*gfp*::*3xFLAG]) I; naSi22 (mex-5P*::*daf-18*::*nos-2 3’UTR*::*SL2*::*GFPo*::*PH*::*tbb-2 3’UTR) II; unc-119(ed3) III*?*; daf-18(ok480) IV*, **GC1627** *glh-1(sam24[glh-1*::*gfp*::*3xFLAG]) I; daf-18; naEx261 (daf-18P*::*daf-18*::*daf-18 3’UTR*::*SL2*::*GFP*::*H2B*::*unc-54 3’UTR)*, **GC1621** *ppw-1 (pk2505) I; naSi2 II*, **GC1390** *rrf-1 (pk1417) I; naSi2 II*, **GC1638** *naSi2 II; unc-119(ed3) III*?*; otIs45 (unc-119*::*GFP) V*, **GC1639** *naSi2 II; unc-119(ed3) III*?*; daf-18(ok480) IV; otIs45 (unc-119*::*GFP) V*.

Transgenic strains were generated by three different methods, as indicated below (see S1 Table):

1. Microinjection (generating extrachromosomal arrays) [82] directly into GC1459 *naSi2 II; daf-18(ok480) IV*. All arrays used pJB134 (*flp-17*P::dsred) as a co-injection marker [83].
2. Cas9-triggered long-range HDR using plasmid repair template, and plasmid Cas9 and single guide RNA [43]. Although the *Phsp*::*peel-1* was included in the injection mix we found that the heat shock step was not necessary (Used to generate *germline*::*daf-18 (naSi22)*).
3. CRISPR-based "hybrid" GFP donor method [84], with minor modifications from J. Nance that used *dpy-10* co-CRISPR instead of *rol-6(su1006d)*. Injection was done into *GC1171 naSi2 II* to generate *GFP*::*vbh-1 (na110)*. crRNA targeting sequence for *vbh-1* and *dpy-10* (co-CRISPR) were generated by IDT. https://www.idtdna.com/site/order/designtool/index/CRISPR_CUSTOM. Cas9 and tracrRNA were purchased from IDT.

### Starved L1 preparation / L1 synchronization

Loosely-synchronized L1s (From small scale embryo preparation).

For some experiments (Images in Fig 1A, tissue-specific *daf-18* “rescue” experiments in Fig 2, a-amanitin experiment in Fig 3E, immunofluorescence in S4 and S6 Figs) a loose synchronization was sufficient. For this protocol, each strain was grown on 1–3 6 cm NGM plates seeded with OP50. Gravid hermaphrodites and embryos were washed with ~1 mL M9 buffer from plates into a 1.8 mL Eppendorf tube. A pure preparation of clean (bacteria-free) embryos was obtained by sodium hypochlorite treatment (1 mL, freshly-prepared, final concentration: 10% sodium hypochlorite solution [Sigma #425044, available chlorine 10–15%], 10% 5N NaOH, 80% M9 buffer). Reactions were monitored under a dissecting microscope and proceeded until no whole adult carcasses remained (~2–5 min). Samples were then washed 5x with ~1 mL M9 buffer (with pelleting at ~1690+ g), yielding purely embryos. Embryos hatched overnight in ~1 mL M9 buffer + 0.08% ethanol in 1.8 mL tubes, with rocking (rocking was omitted for a-amanitin experiment). Therefore, L1 larvae were loosely synchronized with respect to hatching. L1 larvae were assessed at ~24 or ~72 hours from the embryo preparation as noted in figure legends, or in graph titles for Figs 2 and 3E, as “1 Day Starved” or “3 Days Starved”, respectively.

#### Tightly-synchronized L1s (From large scale embryo preparation)

For experiments requiring tight synchronization of large batches of L1 larvae with respect to hatching (experiments: timing of PGC divisions in Fig 1B and 1C, immunofluorescence in Fig 3, and RNA Sequencing in Fig 5), animals were grown on a larger scale for embryo preparation. Each strain was grown on 4–6 high peptone NGM plates (10 cm) seeded with OP50 (starting with 40–60 adult hermaphrodites per plate and growing for 5 days at 20°C). Gravid hermaphrodites and all embryos were washed with 10 mL M9 buffer into 2–3 conical tubes (15mL). Pure preparations of clean (bacteria-free) embryos were obtained by sodium hypochlorite treatment (10 mL, freshly-prepared, final concentration: 10% sodium hypochlorite solution [Sigma #425044, available chlorine 10–15%], 10% 5N NaOH, 80% M9 buffer). Reactions were monitored under a dissecting microscope and proceeded until no whole adult carcasses remained (~2–5 min). Samples were then washed 5x with ~10mL M9 buffer (with pelleting at ~1690 g), yielding purely embryos. Embryos hatched for 2 hours in 10 mL M9 buffer (Immunofluorescence experiments, Fig 3) or M9 + 0.08% ethanol (100%) (timing of PGC divisions in Fig 1B and 1C and RNA Sequencing in Fig 5), in 2–3 conical tubes (15 mL). Resulting L1s were separated from embryos using 10 μm mesh Pluristrainers (Pluriselect # 43-50010-03). L1s were strained into 50 mL conical tubes (and embryos caught in the filter), washed 5x with 5mL M9, with swirling and pipetting up and down to maximize L1 yield and prevent clogging of filter with embryos. Resulting L1s larvae were tightly synchronized, having hatched within the previous 2 hours. To proceed with immunostaining or RNA Sequencing, larvae were pelleted by centrifugation for 2 minutes at 900-1300g (yields ~5 μL or less of compacted L1s, or estimated 20,000–40,000 worms). For RNA Sequencing experiment, pelleted L1 larvae were flash frozen in liquid nitrogen and stored at -80°C until shipping all samples on dry ice for analysis.

#### Tightly-synchronized L1s (from very small scale embryo preparation)

For experiments requiring selection of individual hermaphrodite mothers (Maternal/Zygotic *daf-18* requirement experiments (Fig 1B and 1C) and all zygotic gene expression experiments (Figs 4 and 6 and S4 and S6 Figs), gravid hermaphrodite P0s were selected and then bleached on unseeded NGM plates. Up to 15 hermaphrodites were placed in the center of the plate and treated with ~15μL of 1:1 sodium hypochlorite solution (Sigma, 10–15% available chlorine) and 5N NaOH. Each reaction was monitored under a dissecting microscope, and free embryos were moved out of the bleach drop with a sterile worm pick, while adult carcasses and fragments remained in the bleach. Once all carcass fragments were dissolved and bleach was absorbed into the plate, 15–30μL of M9 buffer was placed on top of embryos as a “wash.” Most embryos had not hatched ~12 hours later, so hatching of remaining embryos could be monitored hourly thereafter (zygotic gene expression experiments) or after several days (maternal/Zygotic *daf-18* requirement experiments).

### Timing of hatching from 2–4 cell stage

Embryos were isolated by dissection of gravid hermaphrodites in M9 in glass wells. Embryos at the 2–4 cell stage were selected under the dissecting microscope with a mouth pipette and transferred to unseeded NGM plates. Embryos were monitored hourly for the number of hatched L larvae and embryos in 4 replicates (2 biological, 2 technical).

### Timing of PGC divisions

Tightly-synchronized L1s were isolated using the large-scale embryo preparation (hatched within a 2 hour time frame, described above). The timing in Fig 1B and 1C are labelled as “Hours post-collection,” meaning that animals examined at time point “0” hatched within the last 2 hours. L1 animals were either left in liquid culture (Fig 1B, “starved”) or plated on NGM plates seeded with OP50 bacteria (Fig 1C, “fed”) for the designated amounts of time (1–4 h).

### PGC counts

L1 larvae were mounted onto 2% or 4% agarose pads on slides, paralyzed with 1mM levamisole (Sigma), and examined at 630x on a Zeiss Z1 Axioimager (with 63x objective) within 1 hour of mounting. PGC number was determined by counting cells positive for *PGC*::*mCherry (naSi2)*, *glh-1*::*GFP* (*glh-1(sam24)*) or membrane *GFP* (*germline*::*daf-18*::*SL2*::*GFP*::*PH*). Dead L1 larvae were always omitted; these were identified by a distinctive auto-fluorescent glow and/or a highly vacuolated appearance.

Embryonic PGCs were counted by mounting *glh-1*::*GFP* (*glh-1(sam24)*)-positive embryos, picked from a plate (mostly 3-fold embryos), onto 2% or 4% agarose pads on slides, in M9 buffer, then examination on a Zeiss Z1 Axioimager (at 630x).

### *daf-18* RNAi

RNAi experiments were conducted as described [85]. Hermaphrodites grown on NGM plates seeded with OP50 bacteria at 20°C were transferred to standard RNAi plates (IPTG/Amp) at the L4 stage. The empty vector L4440 in HT115 *E*. *coli* was the negative control. The efficacy of the RNAi in the various strains was confirmed in parallel by the completely penetrant *skn-1* maternal-lethal phenotype of dead embryos (Emb) in GC1171 *naSi2* and GC1390 *rrf-1; naSi2* but no *skn-1* Emb phenotype for GC1620 *ppw-1; naSi2*. After five days of RNAi feeding (2 generations), pure preparations of clean (bacteria-free) embryos were obtained by sodium hypochlorite treatment of gravid animals on L4440 and *daf-18* RNAi plates. F3 generation worms hatched and were starved for two days in 10 mL of M9 buffer with 0.08% ethanol. Resulting starved L1s were assessed for PGC number. *daf-18* RNAi efficacy was confirmed by inappropriate PGC divisions in starved L1 progeny of GC1171 *naSi2*.

### Immunofluorescence

Strains bearing the PGC marker GLH-1::GFP, fluorescence from which generally survived fixation, were used for all immunofluorescence experiments except for S4 Fig (starved L1 larvae, stained with anti-PGL-1 antibody OIC1D4).

#### Partial dissociation/permeabilization

L1 larvae were first prepared by “Tight Synchronization/Large-scale preparation” described above, which yields a small L1 pellet (~5μL or less of compacted L1 larvae) (for staining experiments in Fig 3). Loosely-synchronized L1 larvae (from overnight hatching of clean, washed embryos prepared by small or large-scale embryo preparation, described above), can also be permeabilized this way. Note that the compacted L1 pellet should be 40μL or less. Larger pellets appear to permeabilize faster, so adjustments to treatment time and strength of mechanical disruption may be required. Similar adjustments may be required for staining of head or tail cells, which are occasionally lost with this permeabilization method.

L1s were chemically and mechanically permeabilized by following an existing L1 cell isolation protocol, which details reagents and recipes for buffers required [86] http://www.wormbook.org/chapters/www_cellculture/cellculture.html)), with minor modifications described below.

Briefly, the permeabilization with modifications was performed as follows (a more detailed protocol is available upon request): The L1 pellet was washed with 1mL ddH_2_O in a low retention Eppendorf tube and re-pelleted at 16,000g for 2 minutes after which as much supernatant as possible was removed with careful pipetting. 200μL of freshly-thawed SDS-DTT solution (20 mM HEPES pH 8.0, 0.25% SDS, 200 mM DTT, 3% sucrose) was added and larvae were incubated for exactly 2 minutes at room temperature, after which 800μL of egg buffer was added and mixed gently. L1 larvae were then pelleted at 16,000g for 1 minute, and washed 5x with egg buffer and gentle mixing; supernatant was again carefully removed. 100μL of freshly-thawed pronase E (15mg/mL, Sigma P8811 in egg buffer) was added and larvae incubated for 7–30 minutes at room temperature. For reasons that are unclear, some strains reliably permeabilized rapidly after 7 minutes of Pronase E treatment, while others required a longer permeabilization of 15–30 minutes, and one strain, GC1456, did not permeabilize even after 30 minutes. During this incubation, a 25-gauge needle, attached to a syringe pump, was used to suck up-and-down the entire worm suspension a few times each minute. Dissociation was monitored every few minutes by placing a drop (1–2 μL) of the reaction onto a slide and analyzing on a dissecting microscope. Once an increase in worm fragments and single cells (which look like dust at 100x) was observed, the reaction was stopped with 900μL L15-FBS (or egg buffer). L1 larvae and fragments were then pelleted for 3 minutes at 1300g, and washed 2x with L15-FBS and gentle mixing. After the last wash, the supernatant was removed, leaving ~100μL of L1 larvae and fragments in buffer.

#### Fixation

For H3K4 antibodies (and anti-PGL-1, OIC1D4, used to co-stain in one experiment), methanol/acetone fixation was conducted in Eppendorf tubes. Fresh aliquots of methanol and acetone were pre-chilled at -20°C. L1 pellet was gently mixed in residual ~100 μL buffer, 1 mL cold methanol was added, and tubes were incubated for 9 minutes at room temperature or -20°C. Samples were centrifuged for 1 minute at 16,000 g and supernatant removed, leaving ~100 μL or less. L1s were resuspended in residual methanol by pipetting and/or brief vortexing (getting as close to single worm suspension as possible to reduce clumping when acetone is added). 1 mL of cold acetone was added, and tubes were incubated for 4 minutes at room temperature or -20°C. Samples were centrifuged for 1 minute at 16,000 g and supernatant removed, leaving ~100 μL or less, then washed 3x with PBS-Tw (PBS with 0.1% Tween). Each wash was 5–10 minutes with rocking, or agitation by hand several times during the incubation.

For H5 staining, methanol/formaldehyde fixation was used. The protocol above was followed, with the following changes: -20°C methanol step was 2 min, followed by formaldehyde fix (1x PBS, 0.08M HEPES (pH 6.9), 1.6 mM MgSO4, 0.8mM EGTA, 3.7% formaldehyde) [87] for 5 minutes at room temperature. Wash buffer was PBS-Tr (PBS with 0.1% Triton-X).

#### Antibody staining

For H3K4me antibodies (and anti-pgl-1 OIC1D4), blocking was with 100 μL (or more) PBS-Tw + 0.1% BSA for 30 min. For H5 staining, blocking was with 100 μL (or more) of 9 parts PBS-Tr + 0.1% BSA to 1 part normal goat serum (NGS) for 30 minutes. 1° antibodies, all diluted in respective blocking buffers and incubated overnight at 4°C, were: CMA303 (mouse anti-H3K4me2, Millipore Sigma, Cat # 05-1338-S, diluted 1:20) [51,88], ab32356 (rabbit anti-H3K4me2, Abcam, diluted 1:250) [89], ab8580 (rabbit anti-H3K4me3, Abcam, diluted 1:500) [90], H5 (mouse anti-P-Ser2 RNA Pol II, Biolegend Cat. # 920201, diluted 1:50), OIC1D4 (mouse anti-PGL-1, Developmental Studies Hybridoma Bank, gift of J. Nance, diluted 1:2), anti-GFP (rat, Nalcalai Tesque 04404–84, diluted 1:1,000), and anti-dsRed (rabbit, Takara Bio/Clontech Cat. # 632496, diluted 1:500).

After 1° antibody incubation, samples were washed for 10 minutes with rocking, 3-5x with PBS-Tw or PBS-Tr. 2° antibodies used, diluted in respective blocking buffers, were: Goat anti-mouse IgG antiserum, Cy3 conjugated (Jackson Labs, Cat. # 115-165-164, diluted 1:200), goat anti-rabbit IgG CY3-conjugated (from Jackson Labs in 2012, Cat. # 111-166-003, diluted 1:200), goat anti-mouse IgM-Alexa Fluor 594 (Invitrogen, Cat. # A-21044, diluted 1:200), and goat anti-rabbit IgG-Alexa Fluor 488 (Invitrogen, Cat. # A-11034, diluted 1:200).

After 2° antibody incubation, samples were washed for 10 minutes with rocking, 3-5x with PBS-Tw or PBS-Tr with DAPI (25 μg/mL) in the second wash.

Stained L1 larvae were mounted on 2% agarose pads with ProLong Glass antifade mounting media, covered with coverslips, and analyzed immediately, or allowed to cure overnight for imaging the next day.

#### Embryo permeabilization/fixation/staining

Embryos in S5A Fig were prepared by freeze cracking gravid adult hermaphrodites [91] on slides, then proceeding to fixation and antibody staining, using the same concentrations of reagents as described above for L1 larvae, but with wash steps performed in Coplin staining jars.

### α-amanitin treatment

L1 larvae carrying a PGC marker (*PGC*::*mCherry (naSi2)* or *glh-1*::*GFP* (*glh-1(sam24)*) were loosely synchronized (using small scale embryo preparation) and treated with 10 μg/mL α-amanitin (Sigma A2263), as in Butuci et al. (2015), during hatching and starvation up to one day. Animals that appeared dead by bright auto-fluorescence throughout the body, or highly vacuolated appearance, were omitted. To confirm that death caused by the drug was not skewing PGC counts, for 2–3 of the five replicates (wild-type and *daf-18*, respectively), the number of dead L1 larvae were recorded +/- α-amanitin; little to no effect was seen (S7 Fig).

### Maternal and zygotic *daf-18* requirements

#### Maternal- Zygotic+

*cdc-42*::*GFP* was crossed in from the male (P0), as a marker to identify cross- versus self-progeny, along with a copy of the *daf-18(+)* gene (genomic, wild type). Hermaphrodite mothers of the cross (P0) were *naSi2 (PGC*::*mCherry); daf-18(ok480)*. Animals mated for ~24 hours on NGM seeded with OP50 “cross plates” (small bacterial lawn). Mated hermaphrodites were treated with sodium hypochlorite on unseeded NGM plates (Very Small Scale L1 synchronization, described above) to isolate embryos free of bacteria. Progeny (starved L1 larvae) were scored 3 days later and for numbers of PGCs and presence or absence of *cdc-42*::*GFP* (inferred *daf-18* (+) positive/negative).

#### Maternal+ zygotic-

*daf-18(0)* gravid hermaphrodite mothers carrying a *daf-18(+)* genomic rescuing array (*naSi2; daf-18(ok480); naEx261 (daf-18P*::*daf-18*::*SL2*::*GFP)*+) were selected by fluorescence (both *daf-18 GFP* positive and co-injection/transformation marker, *flp-17P*::*dsRed* positive). These worms were treated with sodium hypochlorite on an unseeded NGM agar plate (Very Small Scale L1 synchronization, described above) to isolate embryos free of bacteria. Resulting L1 worms (starved) were assessed 3 days later, scoring for the number of PGCs and presence or absence of the rescuing transgene (only animals lacking both the transformation marker and *daf-18 GFP* were counted as negative).

### Zygotic gene expression

Males carrying homozygous fluorescently-tagged genes *vbh-1 (na110 [GFP*::*vbh-1]*), *glh-1 (sam24 [glh-1*::*GFP]*), *naSi2 [mex-5P*::*mCherry]*, and *otIs45 [unc-119P*::*GFP]*) were generated by heat shock, and populations were expanded and maintained by crossing to hermaphrodites of the same strain. 2–4 crosses were performed per strain, in both the *daf-18(+)* and *daf-18* mutant backgrounds, with 25–30 males and 8–10 hermaphrodites per cross. Hermaphrodites carried an alternate-color PGC marker (e.g. *glh-1*::*GFP* males crossed to *mex-5*::*mCherry* hermaphrodites). P0 animals mated for 24 hours on NGM plates with small lawns of OP50, and then mated hermaphrodites were treated with sodium hypochlorite on unseeded NGM plates to isolate clean embryos (Very small scale embryo preparation).

The resulting embryos were handled in two different ways. For “short” starvation, comparing wild type and *daf-18* mutants (Figs 4 and 6 and S8 Fig), L1 larvae were very tightly synchronized and starved for ≤5 hours: embryos (mostly 3-fold stage) were moved to fresh unseeded NGM plates (by mouth pipetting with M9 buffer under a dissecting microscope) 12–16 hours after sodium hypochlorite treatment of mothers. Resulting starved L1 larvae were removed (also by mouth pipetting) 1–5 hours after hatching and analyzed for fluorescent zygotic gene expression (see **Microscopy and Image Analysis**, *For zygotic gene expression experiments* below). For “fed” wild type we included an additional control of starved counterparts (“starved”) to ensure that feeding-induced transgene expression is a function of feeding and not simply a function of time. We prepared and imaged the “fed” and “starved” worms in parallel as follows. After clean mixed-stage embryos (from the sodium hypochlorite treatment of mothers) were allowed to hatch 12–19 hours on unseeded NGM plates, resulting starved L1 larvae were then moved (by mouth pipetting) to OP50 bacterial lawns on NGM plates (for “fed”) or left on unseeded plates (for “starved” controls) for 5 hours before analysis.

For zygotic gene expression experiments utilizing RNAi, methods were the same as described above, except: crosses were performed on standard RNAi plates (IPTG/Amp) seeded with small lawns of RNAi bacteria (HT115 carrying plasmids for dsRNA production) [85,92]. Hermaphrodites fed on RNAi bacteria starting from the L4 stage until 24 hours later (adulthood), and were then treated with sodium hypochlorite on NGM plates to isolate clean embryos (Very small scale embryo preparation). The negative control was the empty vector (L4440). *let-363* RNAi efficacy was confirmed by suppression of *daf-18* PGC divisions in starved L1 progeny, and 100% larval (L3) arrest in progeny from crosses that had continued feeding on RNAi plates (Fig 6). RNAi efficacy for additional experiments (2 replicates for *GFP*::*vbh-1*, one replicate for *glh-1*::*GFP*) was confirmed only by 100% larval arrest in RNAi-fed progeny.

### Microscopy and image analysis

Imaging was performed with a Zeiss Z1 AxioImager equipped with an AxioCamMRm digital camera, ApoTome for optical sectioning, a plan apo 63X/1.4 oil DIC objective, and Zeiss filters for DAPI, CY3, and eGFP.

#### For tissue-restricted daf-18 GFP expression

Tissue expression of *daf-18 GFP* transgenes in L1 larvae starved for 1–3 days was documented by taking epifluorescence image stacks of animals mounted on 2% agarose pads, paralyzed with 1mM levamisole. Images are shown as Z-projections (S2 Fig).

#### For immunofluorescence experiments

Fluorescence image stacks were taken using Apotome for sectioning (63x objective, ~0.3μm slices, or optimal slice for fluorophore/filter). For Fig 3 and S4 Fig (WT vs. *daf-18*), exposure times were determined by wild-type stained animals and held constant for all samples and replicate experiments. For S6 Fig, since overall fluorescence levels varied between worms and between experiments, exposure times were adjusted for each experiment; however, quantification was internally controlled (see below, PGC/somatic ratio). Mean fluorescence was quantified with Fiji/ImageJ by drawing an ellipse around each PGC (identified by PGC marker GLH-1::GFP, *anti-pgl-1* co-staining, anti-GFP of an encoded germline::GFP, or DIC), in the image slice where it (based on surrounding P granules (GLH-1::GFP)) was most in focus. The nearest somatic cell/somatic gonad precursor (SGP) mean fluorescence. In addition, background within the worm but outside of nuclei, was measured in the same image. PGC and SGP fluorescence values were corrected for background fluorescence, and PGC/SGP ratio was calculated. When two PGC/somatic values could be calculated for the same animal (cases where both PGCs and surrounding somatic cells showed staining), they were measured separately with the cognate SGP and the ratios were averaged.

#### For zygotic gene expression experiments

L1 larvae were mounted on 4% agarose pads containing 10-100mM levamisole in M9. Higher levamisole concentrations were used for zygotic transgene expression requiring longer exposure times (*naSi2 [PGC*::*mCherry]*). GFP, mCherry, and DIC channels were collected for each animal, within 1 hour of mounting. Exposure times were held constant for each individual experiment but varied based on zygotic transgene. When possible, both PGCs were imaged for each animal and values were averaged, resulting in a single fluorescence value for each animal. If one PGC was obscured (e.g., by intestine/auto-fluorescent gut granules or other cells), it was omitted, and only one PGC was imaged (Figs 4 and 6).

To analyze perinuclear GFP::VBH-1 or GLH-1::GFP expression, a script for Fiji/ImageJ was written by Michael Cammer (NYU Langone Microscopy Laboratory). This allowed identification of PGC nuclei by manual thresholding of each image in the red channel (PGC::mCherry), and automatic measurement of mean GFP channel fluorescence intensity of a 1μm-wide ring surrounding the nucleus.

For zygotic *naSi2* (*PGC*::*mCherry*) expression, regions of interest were hand drawn around PGC nuclei and mean fluorescence measured.

For *unc-119P*::*GFP* head fluorescence measurements, ellipsoid regions of interest were drawn over a single neuron in the head (dorsal, just posterior to the nerve ring, likely a chemosensory neuron) and a single tail neuron near the rectum, and mean fluorescence was measured. The lack of any visible *unc-119P*::*GFP* in PGCs (even upon long exposure times) was noted in all animals that were imaged and measured for head fluorescence.

For all zygotic transgene expression analyses, background fluorescence was measured for each animal in a region within the worm but outside of fluorescent tissues, and was subtracted from zygotic fluorescence-of-interest mean values.

## RNA sequencing experiment

### GC1171 (*naSi2, PGC::mCherry*) versus GC1459 (*naSi2, PGC::mCherry; daf-18 (ok480)*)

#### RNA isolation and RNA-seq library preparation

RNA was isolated using TRIzol Reagent (Thermo-Fisher Scientific) following the manufacturer’s protocol with exceptions noted below. The procedure was scaled down in a linear fashion, using only 100 uL Trizol. 5 ug linear polyacrylamide (Sigma-Aldrich) was included as a neutral carrier for RNA precipitation. RNA was eluted in nuclease-free water and incubated at 55°C for approximately four minutes to resuspend the pellet. 140 ng of total RNA was used for each library preparation. NEBNext Ultra II RNA Library Prep kit (New England Biolabs) was used to perform poly-A selection and prepare libraries for sequencing. The final libraries were enriched with 11 cycles of PCR. Libraries were then sequenced using Illumina NovaSeq 6000 to obtain 50 bp paired-end reads.

#### Differential expression analysis of RNA-seq data

Version WS273 of the *C*. *elegans* genome was used to map sequencing reads (file name c_elegans.PRJNA13758.WS273.genomic.fa downloaded from WormBase). Bowtie version 1.2.2 [93] was used to map 50 bp paired-end reads with the following settings: bowtie -I 0 -X 500 -k 1 -m 2 -S -p 2. Mapping efficiency and number of mapped reads can be found as part of S1 Data. HTSeq version 0.11.2 [94] was used to count reads mapping to the WS273 canonical geneset (file name c_elegans.PRJNA13758.WS273.canonical_geneset.gtf downloaded from WormBase). edgeR version 3.24.3 [95] was used for differential expression analysis. Count data was restricted to include only protein-coding genes (20,127 genes). Prior to differential expression analysis, this list was restricted to include only genes with counts-per-million (CPM) greater than one in at least four libraries (12,592 genes). Principal component analysis (PCA) was performed on log_2_ mean normalized CPM values for the genes included in differential expression analysis. PCA separates libraries based on strain (S9 Fig). The Pearson correlation coefficient for each pair of libraries was calculated using log_2_ normalized CPM values for genes included in differential expression analysis. All replicate libraries have a Pearson correlation coefficient of 0.98–0.99 (S9 Fig). In edgeR, the “calcNormFactors” function was used to normalize for RNA composition and the tagwise dispersion estimate was used for differential expression analysis. The exact test was implemented in edgeR for pairwise comparison of four biological replicates of GC1171 to four biological replicates of GC1459. 3,335 genes are differentially expressed at an FDR cutoff of 0.05. All edgeR differential expression output, including gene name, log_2_ fold change, expression level, and FDR, can be found as part of S1 Data.

#### Gene group analysis for RNA-seq

We collected several independent lists of genes previously reported to be enriched or expressed in the germ line:

1. 1346 genes enriched in sorted, labeled embryonic primordial germ cells, Z2 and Z3, over somatic blastomere genes (Supplementary File 8 from Lee et al., 2017)
2. 979 genes enriched in sorted, labeled embryonic primordial germ cells, Z2 and Z3, over all embryonic cells (Supplemental File Z2/Z3 #2 from Spencer et al., 2011)
3. 306 genes 5x enriched in the L2 germline [45], data accessed by GExplore1.4 web tool for tissue expression [67]
4. 4980 genes expressed in embryonic primordial germ cells, Z2/Z3, for which all three “pseudotime” bins are above 0 TPM.

To determine whether gene groups of interest [41,45,58,65] tend to be up- or down-regulated in the *daf-18* mutant compared to wild-type, the cumulative distribution of log_2_ fold changes for each group of genes in the GC1459 / GC1171 comparison was plotted along with the cumulative distribution of log_2_ fold changes of all 12,592 detected genes in the GC1459 / GC1171 comparison. Differences between the two cumulative distributions were assessed using the Kolmogorov-Smirnov test. In a complementary approach, the overlap between each gene group of interest and differentially expressed genes that were up- or down-regulated in the GC1459 / GC1171 comparison (FDR < 0.05) was assessed, and the hypergeometric test was used to determine significant overlap. Gene groups used in this analysis can be found in S1 Data. Finally, tissue enrichments and GO terms were assessed among genes up- and down-regulated in GC1459 / GC1171 using the WormBase Gene Set Enrichment Analysis tool [63,64].

## Supporting information

S1 Fig *daf-18* mutant embryos hatch at the same time as wild type. The number of L1 larvae hatched from embryos isolated at the 2–4 cell stage was monitored hourly. 4 replicate experiments (2 biological, 2 technical), containing a total of 40 WT (*PGC*::*mCherry*, *naSi2*) animals, and 49 *daf-18* mutant animals (*naSi2; daf-18*).
(TIF)

S2 Fig Expression patterns of *daf-18(+)*::*GFP* constructs. (A-G) All animals are *daf-18(ok480)* mutants, and all carry *PGC*::*mCherry* (*naSi2*), except for panel (B). Scale bars represent 10μm, white arrows indicate GFP expression in expected tissues-of-interest, pink arrows indicate PGCs, and white stars indicate a transgenic co-injection marker (*flp-17P*::*dsRed*) which was used to identify all extrachromosomal array-bearing animals. All *daf-18(+)* constructs (various promoters driving *daf-18(+)*::*SL2*::*GFP*::*H2B*) are carried as extrachromosomal arrays (with nuclear GFP for cell identification), except for *germline*::*daf-18(+)* (in panel (B)), which is a single copy insertion. Arrays carry the *daf-18* 3’UTR on the *daf-18* rescuing portion, and the *unc-54* 3’UTR for the GFP reporter portion to allow broad somatic expression [96] (e.g. *rab-3P*::*daf-18(+)*::*daf-18 3’UTR*::*SL2*::*GFP*::*H2B*::*unc-54 3’UTR*). (B) The *germline*:: *daf-18(+)* construct (*mex-5P*::*SV40 NLS*::*daf-18 coding region*::*nos-2 3’UTR*::*SL2*::*GFPo*::*PH*::*tbb-2 3’UTR)* has several features. First, the GFP is fused to a PH domain thereby targeting the GFP to the cell membrane. Second, there are two different 3’ UTRs, one following the *daf-18* portion and another following the GFP. Specifically, the *nos-2* 3’UTR follows the *daf-18* rescuing portion (as used in our *PGC*::*mCherry*, seen in the other panels of this figure), which restricts expression to the PGCs (1 additional head cell is marked in ~10% of animals). The *tbb-2* 3’UTR was used for the GFP portion, which allows somewhat broader expression as is visible in somatic tissues. Nevertheless, *daf-18(+)* expression should be limited to PGCs, as evidenced by the *PGC*::*mCherry* reporter *naSi2* which expresses mCherry from the exact same *mex-5* promoter and the same *nos-2 3’* sequence. Last, an NLS is fused in frame with DAF-18; it is possible that it enhances DAF-18 nuclear activity. (H) Approximate embryonic transcript levels [41], in germline and somatic tissues of interest, of genes for which promoters were used to drive tissue-specific expression. *Adjusted transcripts per million (TPM), **Germline expression is an approximate range (from Germline:pseudotime bin 1, 2, and 3), ***Approximate range of highest-expressing cells within tissue of interest, ****Approximate range in chemosensory neurons, # Approximate range in intestine, $ Approximate range in somatic cells (neurons, body wall muscle, intestine, other).
(TIF)

S3 Fig Tissue-restricted RNAi is consistent with germline DAF-18 as a regulator of PGC quiescence. F3 progeny of animals fed HT115 bacteria expressing control (Ctrl), empty vector L4440, or *daf-18* dsRNA (feeding from P0 to F2) were assessed for number of PGCs after 2 days of starvation as L1s. All strains carry *naSi2* (*PGC*::*mCherry*). n values display the total number of L1 larvae examined for each genotype across three biological replicates in which all three strains were subject to RNAi in parallel in each replicate. Two-sided Fisher’s exact tests were performed to compare within genotype and statistical significance is displayed (ns p>0.05, ****p<0.0001).
(TIF)

S4 Fig Marks of active transcription are inappropriately elevated in the PGCs of starved *daf-18* mutants (additional L1 time points, and with an additional antibody). (A,B). Mean staining fluorescence was measured for each PGC nucleus and its nearest somatic cell nucleus (from single image slices), and is displayed in the graph as a ratio. (A) Antibody ab32356 (H3K4me2) was used to stain wild type (WT) and *daf-18(ok480)* mutant L1s, starved 6–8 hours after hatching (synchronized within 2 hours of hatching and imaged 6 hours later). PGCs were identified with anti-PGL-1 antibody (OIC1D4). Each dot represents a single PGC/somatic cell pair (sometimes 2 values per animal). (B) Antibody ab8580 (H3K4me3) was used to stain WT and *daf-18(ok480)* mutant L1s hatched and starved overnight (~16h). PGCs were identified with encoded *glh-1*::*GFP*. Each dot represents one value per worm (2 PGC/somatic values averaged). Statistical significance determined by two-tailed T-test. **p<0.01, ****p<0.0001.
(TIF)

S5 Fig Signs of inappropriate transcriptional activity in PGCs of *daf-18* mutants may begin late in embryogenesis. (A) Antibody ab32356 (anti-H3K4me2) was used to stain wild type (WT) and *daf-18(ok480)* mutant 3-fold embryos. PGCs were identified with *glh-1*::*GFP*. Mean fluorescence was measured for each PGC and its nearest somatic cell (from single image slices), and is displayed in the graph as a ratio. Each dot is a single PGC/somatic cell pair. Statistical significance determined by two-tailed T-test. ***p<0.001. (B) and (C) Zygotic expression of GFP::VBH-1 was examined by epifluorescence (as in Fig 4) in embryonic PGCs. (B) Mean perinuclear GFP fluorescence intensity (AU = Arbitrary Units) values are plotted. Graph shows results from three separate experiments pooled. Each dot represents one animal; colored dots correspond to images in panel (C). (C) Images of the brightest perinuclear GFP::VBH-1, in PGCs of WT and *daf-18* mutants, according to quantification. Each arrowhead points to perinuclear GFP of one PGC. Gut granules (green autofluorescent dots) are marked with asterisks. Scale bars represent 10μm.
(TIF)

S6 Fig Active chromatin marks are low in PGCs expressing DAF-18 during starvation. Antibodies ab8580 (anti-H3K4me3) (A, B) and CMA303 (anti-H3K4me2) (C, D) were used to stain L1 larvae starved for up to 24 hours. Tricolor images (left panels) show DNA (DAPI/blue), histone mark immunofluorescence (green), and either a PGC marker (see below) or transgenic animal marker (magenta). Maximum intensity projections of image stacks are shown, except for (A) (top panels), which show a single slice. Dashed circle indicates one PGC in each image, and dashed smaller ellipse indicates its nearest somatic cell (likely its SGP). White arrowheads point to PGCs, and green arrowheads point to neighboring somatic cells. Scale bars represent 10μm. Mean staining fluorescence was quantified for each PGC nucleus and its nearest somatic cell nucleus (from single image slices), and is displayed in the correlated graph as a ratio. Each dot in the graph represents one animal (2 PGC/SGP values averaged or a single PGC/SGP pair). Dot colors represent data gathered on different days / in different experiments. Triangles represent animals shown in the images, and open circles represent animals with 3 or more PGCs. Statistical significance determined by one-way ANOVA. **p<0.01, ***p<0.001. (A) Magenta marks germ cell membranes (anti-GFP in L1 larvae carrying *germline*::*daf-18*::*SL2*::*GFP*::*PH*) (top panels) or intestinal cells (anti-GFP in L1s carrying *intestine*::*daf-18*::*SL2*::*GFP*::*H2B*) (bottom panels). PGCs were identified by anti-GFP membrane marker (top) or DIC (bottom). Transgenic animals carrying daf-18(+) rescuing arrays were identified by anti-GFP. (B) “*daf-18* control*” represents non-transgenic siblings of *daf-18; intestine*::*daf-18(+)* animals in the middle column (black dots), and a mix of transgenic and non-transgenic *daf-18; neurons*::*daf-18(+)* (blue dots). Since these are F3 progeny of P0 mothers that were selected (transgene-positive), possible maternal contributions of transgenes are unknown.(C) Magenta marks germ cell P granules (*glh-1*::*GFP*, falsely colored, bottom panels) to identify PGCs. PGCs were also identified by DIC (top panels). Transgenic animals in *daf-18; daf-18 genomic (+)* were identified by anti-dsRed, which stains an encoded *flp-17P*::*dsRed* coinjection marker, expressed in a pair of head neurons. (D) “daf-18 control*” represents non-transgenic siblings of *daf-18; daf-18 genomic(+)* animals in the middle column. Since these are F3 progeny of P0 mothers that were selected (transgene-positive), possible maternal contributions of transgenes are unknown.
(TIF)

S7 Fig α-amanitin does not affect survival of L1 larvae starved up to 1 day. Embryos (from a bleach preparation) carrying a PGC marker (*naSi2 PGC*::*mCherry* or *sam24 glh-1*::*GFP*) were allowed to hatch and starve up to 24h, with or without 10ug/mL α-amanitin, before assessing PGC numbers (see Fig 3E) and dead/total L1 larvae. Colors represent different replicates. Line indicates the median.
(TIF)

S8 Fig Further zygotic transgene expression analysis. (A-C) Images show epifluorescence. Mean fluorescence intensity (AU = Arbitrary Units) values are plotted to the right of each set of images. Each dot represents one animal. Non-cross progeny were identified by the lack of somatic GFP ((A) and (C)) or lack of PGC::mCherry (B), which are all visible when present, even in starved animals. Mean +/- SEM. All scale bars represent 10μm. (A) Zygotic GFP::VBH-1 expression in the soma (head neurons) is not expressed more highly in starved *daf-18* mutants than starved wild type (WT). Dashed ellipses show regions measured for mean fluorescence. As in Fig 4, “Starved (short)” animals were imaged within 5 hours of hatching. “Starved” progeny were imaged up to 22 hours after clean embryo preparation, while “fed” L1 progeny were imaged at the same time, but after 5 hours of feeding (after up to 17 hours post-embryo preparation). Graph shows the pooled results of 2 experiments. Statistical significance determined by one-way ANOVA with Tukey’s multiple comparisons test. ***p<0.001. (B) Zygotic nuclear PGC::mCherry expression is not expressed more highly in starved *daf-18* mutant PGCs than starved WT PGCs. L1s were imaged up to 19 hours after clean embryo preparation. Arrows point to zygotic PGC::mCherry expression in PGCs, and asterisks mark auto-fluorescent gut granules. Nuclear PGC::mCherry fluorescence was measured by hand-drawing regions of interest around PGC nuclei. One experimental replicate is graphed, and 2 others showed similar results. Statistical significance determined by one-way ANOVA with Tukey’s multiple comparisons test. (C) Zygotic *unc-119P*::GFP is not expressed in the PGCs of starved *daf-18* mutants. However, it is expressed at a slightly higher level in neurons of starved *daf-18* mutants. L1s were imaged up to 18 hours after clean embryo preparation. Dashed circles indicate approximate neuronal regions measured (one head neuron and one tail neuron per animal). Graph shows the pooled results of 2 experiments. Statistical significance determined by two-tailed T-test. *p<0.05.
(TIF)

S9 Fig RNA Sequencing: Quality control analyses. (A) Principal component analysis (PCA) of GC1171 (wild type) and GC1459 (*daf-18(ok480)*) starved L1s in RNA Sequencing experiment, highlighting low variance within each genotype and substantial variance between genotypes. (B) Correlation matrix of GC1171 (wild type) and GC1459 (*daf-18(ok480)*) showing that replicates of the same genotype were highly correlated.
(TIF)

S10 Fig RNA sequencing further germline gene analyses. (A) Venn diagram showing the overlap of all four germline gene sets analyzed [41,45,58,65], and numbers of genes from these sets detected in our RNA Sequencing experiment. (B-E) Cumulative distribution function (CDF) plots for individual published germline gene sets (red lines) compared to the background set of all transcripts detected in our experiment (12,592 genes). p-values determined by Kolmogorov-Smirnov (KS) test are shown. Numbers of transcripts from published gene sets detected in our experiment are shown in graph titles (parentheses).
(TIF)

S11 Fig Fed versus starved L1 expression among the “set of 42” germline genes (data from Baugh et al., 2009). Individual Z-scores of these 40 genes (those detected in this dataset out of 42 identified in Fig 5E) are plotted over time, and confidence intervals (95%) of their average are plotted in grey.
(TIF)

S12 Fig RNA Sequencing: Analyses of tissues and GO terms. Cumulative distribution function (CDF) plots of transcript sets of interest (red line) versus the background set of all 12,592 transcripts detected (black line). p-values determined by Kolmogorov-Smirnov (KS) test are shown. Numbers of transcripts from published gene sets detected in our experiment are shown in graph titles (parentheses). (A-F) CDF plots for lists of transcripts from tissues of interest known to express *daf-18* (ciliated neurons, non-seam hypodermis, seam cells, body wall muscle), and tissues that may not express *daf-18* (touch receptor neurons, pharyngeal gland) [41,45]. Tissue-specific gene lists were generated using the GExplore1.4 web tool (underlying data from Cao et al., 2017). (G-N) CDF plots for lists of transcripts associated with specific gene ontology (GO) terms (using WormBase). (O) Unbiased analysis of gene ontology (GO) terms, using WormBase’s Enrichment Analysis Tool.
(TIF)

S1 Table Strains, plasmids and primers used in this study. (XLSX)

S1 Data RNA-sequencing results and analysis. (XLSX)

## References

[1] Cheung TH, Rando TA. Molecular regulation of stem cell quiescence. Nat Rev Mol Cell Biol. 2013;14(6):329–40. doi: 10.1038/nrm3591 23698583 PMC3808888

[2] Coller HA. The paradox of metabolism in quiescent stem cells. FEBS Lett. 2019;593(20):2817–39. doi: 10.1002/1873-3468.13608 31531979 PMC7034665

[3] Shyh-Chang N, Daley GQ, Cantley LC. Stem cell metabolism in tissue development and aging. Development. 2013;140(12):2535–47. doi: 10.1242/dev.091777 23715547 PMC3666381

[4] Coller HA. Regulation of Cell Cycle Entry and Exit: A Single Cell Perspective. Compr Physiol. 2019;10(1):317–44. doi: 10.1002/cphy.c190014 31853969 PMC8208229

[5] Fukuyama M, Rougvie AE, Rothman JH. C. elegans DAF-18/PTEN mediates nutrient-dependent arrest of cell cycle and growth in the germline. Curr Biol. 2006;16(8):773–9. doi: 10.1016/j.cub.2006.02.073 16631584

[6] Nguyen PD, Gurevich DB, Sonntag C, Hersey L, Alaei S, Nim HT, et al. Muscle Stem Cells Undergo Extensive Clonal Drift during Tissue Growth via Meox1-Mediated Induction of G2 Cell-Cycle Arrest. Cell Stem Cell. 2017;21(1):107–19 e6. doi: 10.1016/j.stem.2017.06.003 28686860

[7] Otsuki L, Brand AH. Cell cycle heterogeneity directs the timing of neural stem cell activation from quiescence. Science. 2018;360(6384):99–102. doi: 10.1126/science.aan8795 29622651 PMC6538531

[8] van Velthoven CTJ, Rando TA. Stem Cell Quiescence: Dynamism, Restraint, and Cellular Idling. Cell Stem Cell. 2019;24(2):213–25. doi: 10.1016/j.stem.2019.01.001 30735649 PMC6413865

[9] Batlle E, Clevers H. Cancer stem cells revisited. Nat Med. 2017;23(10):1124–34. doi: 10.1038/nm.4409 28985214

[10] Cho IJ, Lui PP, Obajdin J, Riccio F, Stroukov W, Willis TL, et al. Mechanisms, Hallmarks, and Implications of Stem Cell Quiescence. Stem Cell Reports. 2019;12(6):1190–200. doi: 10.1016/j.stemcr.2019.05.012 31189093 PMC6565921

[11] Pavlova NN, Thompson CB. The Emerging Hallmarks of Cancer Metabolism. Cell Metab. 2016;23(1):27–47. doi: 10.1016/j.cmet.2015.12.006 26771115 PMC4715268

[12] Hill R, Wu H. PTEN, stem cells, and cancer stem cells. J Biol Chem. 2009;284(18):11755–9. doi: 10.1074/jbc.R800071200 19117948 PMC2673242

[13] Sun H, Lesche R, Li DM, Liliental J, Zhang H, Gao J, et al. PTEN modulates cell cycle progression and cell survival by regulating phosphatidylinositol 3,4,5,-trisphosphate and Akt/protein kinase B signaling pathway. Proc Natl Acad Sci U S A. 1999;96(11):6199–204. doi: 10.1073/pnas.96.11.6199 10339565 PMC26859

[14] Kimura T, Suzuki A, Fujita Y, Yomogida K, Lomeli H, Asada N, et al. Conditional loss of PTEN leads to testicular teratoma and enhances embryonic germ cell production. Development. 2003;130(8):1691–700. doi: 10.1242/dev.00392 12620992

[15] Yilmaz OH, Valdez R, Theisen BK, Guo W, Ferguson DO, Wu H, et al. Pten dependence distinguishes haematopoietic stem cells from leukaemia-initiating cells. Nature. 2006;441(7092):475–82. doi: 10.1038/nature04703 16598206

[16] Zhang J, Grindley JC, Yin T, Jayasinghe S, He XC, Ross JT, et al. PTEN maintains haematopoietic stem cells and acts in lineage choice and leukaemia prevention. Nature. 2006;441(7092):518–22. doi: 10.1038/nature04747 16633340

[17] White AC, Khuu JK, Dang CY, Hu J, Tran KV, Liu A, et al. Stem cell quiescence acts as a tumour suppressor in squamous tumours. Nat Cell Biol. 2014;16(1):99–107. doi: 10.1038/ncb2889 24335650 PMC3874399

[18] Worby CA, Dixon JE. Pten. Annu Rev Biochem. 2014;83:641–69. doi: 10.1146/annurev-biochem-082411-113907 24905788

[19] Duan S, Yuan G, Liu X, Ren R, Li J, Zhang W, et al. PTEN deficiency reprogrammes human neural stem cells towards a glioblastoma stem cell-like phenotype. Nat Commun. 2015;6:10068. doi: 10.1038/ncomms10068 26632666 PMC4686761

[20] Leslie NR, Downes CP. PTEN function: how normal cells control it and tumour cells lose it. Biochem J. 2004;382(Pt 1):1–11. doi: 10.1042/BJ20040825 15193142 PMC1133909

[21] Keniry M, Parsons R. The role of PTEN signaling perturbations in cancer and in targeted therapy. Oncogene. 2008;27(41):5477–85. doi: 10.1038/onc.2008.248 18794882

[22] Kalaany NY, Sabatini DM. Tumours with PI3K activation are resistant to dietary restriction. Nature. 2009;458(7239):725–31. doi: 10.1038/nature07782 19279572 PMC2692085

[23] Curry NL, Mino-Kenudson M, Oliver TG, Yilmaz OH, Yilmaz VO, Moon JY, et al. Pten-null tumors cohabiting the same lung display differential AKT activation and sensitivity to dietary restriction. Cancer Discov. 2013;3(8):908–21. doi: 10.1158/2159-8290.CD-12-0507 23719831 PMC3743121

[24] Riddle DL, Swanson MM, Albert PS. Interacting genes in nematode dauer larva formation. Nature. 1981;290(5808):668–71. doi: 10.1038/290668a0 7219552

[25] Vowels JJ, Thomas JH. Genetic analysis of chemosensory control of dauer formation in Caenorhabditis elegans. Genetics. 1992;130(1):105–23. 1732156 10.1093/genetics/130.1.105 PMC1204785

[26] Ogg S, Ruvkun G. The C. elegans PTEN homolog, DAF-18, acts in the insulin receptor-like metabolic signaling pathway. Mol Cell. 1998;2(6):887–93. doi: 10.1016/s1097-2765(00)80303-2 9885576

[27] Narbonne P, Roy R. Inhibition of germline proliferation during C. elegans dauer development requires PTEN, LKB1 and AMPK signalling. Development. 2006;133(4):611–9. doi: 10.1242/dev.02232 16407400

[28] Tenen CC, Greenwald I. Cell Non-autonomous Function of daf-18/PTEN in the Somatic Gonad Coordinates Somatic Gonad and Germline Development in C. elegans Dauer Larvae. Curr Biol. 2019;29(6):1064–72 e8. doi: 10.1016/j.cub.2019.01.076 30827916 PMC6486834

[29] Solari F, Bourbon-Piffaut A, Masse I, Payrastre B, Chan AM, Billaud M. The human tumour suppressor PTEN regulates longevity and dauer formation in Caenorhabditis elegans. Oncogene. 2005;24(1):20–7. doi: 10.1038/sj.onc.1207978 15637588

[30] Baugh LR, Sternberg PW. DAF-16/FOXO regulates transcription of cki-1/Cip/Kip and repression of lin-4 during C. elegans L1 arrest. Curr Biol. 2006;16(8):780–5. doi: 10.1016/j.cub.2006.03.021 16631585

[31] Fukuyama M, Kontani K, Katada T, Rougvie AE. The C. elegans Hypodermis Couples Progenitor Cell Quiescence to the Dietary State. Curr Biol. 2015;25(9):1241–8. doi: 10.1016/j.cub.2015.03.016 25891400

[32] Fukuyama M, Sakuma K, Park R, Kasuga H, Nagaya R, Atsumi Y, et al. C. elegans AMPKs promote survival and arrest germline development during nutrient stress. Biol Open. 2012;1(10):929–36. doi: 10.1242/bio.2012836 23213370 PMC3507181

[33] Kasuga H, Fukuyama M, Kitazawa A, Kontani K, Katada T. The microRNA miR-235 couples blast-cell quiescence to the nutritional state. Nature. 2013;497(7450):503–6. doi: 10.1038/nature12117 23644454

[34] Zheng S, Qu Z, Zanetti M, Lam B, Chin-Sang I. C. elegans PTEN and AMPK block neuroblast divisions by inhibiting a BMP-insulin-PP2A-MAPK pathway. Development. 2018;145(23). doi: 10.1242/dev.166876 30487179

[35] Hong Y, Roy R, Ambros V. Developmental regulation of a cyclin-dependent kinase inhibitor controls postembryonic cell cycle progression in Caenorhabditis elegans. Development. 1998;125(18):3585–97. 9716524 10.1242/dev.125.18.3585

[36] Roy D, Kahler DJ, Yun C, Hubbard EJA. Functional Interactions Between rsks-1/S6K, glp-1/Notch, and Regulators of Caenorhabditis elegans Fertility and Germline Stem Cell Maintenance. G3 (Bethesda). 2018;8(10):3293–309. 30126834 10.1534/g3.118.200511 PMC6169383

[37] Kelly WG, Fire A. Chromatin silencing and the maintenance of a functional germline in Caenorhabditis elegans. Development. 1998;125(13):2451–6. 9609828 10.1242/dev.125.13.2451 PMC4084878

[38] Brisbin S, Liu J, Boudreau J, Peng J, Evangelista M, Chin-Sang I. A role for C. elegans Eph RTK signaling in PTEN regulation. Dev Cell. 2009;17(4):459–69. doi: 10.1016/j.devcel.2009.08.009 19853560

[39] Masse I, Molin L, Billaud M, Solari F. Lifespan and dauer regulation by tissue-specific activities of Caenorhabditis elegans DAF-18. Dev Biol. 2005;286(1):91–101. doi: 10.1016/j.ydbio.2005.07.010 16153634

[40] Nakdimon I, Walser M, Frohli E, Hajnal A. PTEN negatively regulates MAPK signaling during Caenorhabditis elegans vulval development. PLoS Genet. 2012;8(8):e1002881. doi: 10.1371/journal.pgen.1002881 22916028 PMC3420937

[41] Packer JS, Zhu Q, Huynh C, Sivaramakrishnan P, Preston E, Dueck H, et al. A lineage-resolved molecular atlas of C. elegans embryogenesis at single-cell resolution. Science. 2019;365(6459). doi: 10.1126/science.aax1971 31488706 PMC7428862

[42] Suzuki Y, Han M. Genetic redundancy masks diverse functions of the tumor suppressor gene PTEN during C. elegans development. Genes Dev. 2006;20(4):423–8. doi: 10.1101/gad.1378906 16481471 PMC1369044

[43] Dickinson DJ, Ward JD, Reiner DJ, Goldstein B. Engineering the Caenorhabditis elegans genome using Cas9-triggered homologous recombination. Nat Methods. 2013;10(10):1028–34. doi: 10.1038/nmeth.2641 23995389 PMC3905680

[44] D’Agostino I, Merritt C, Chen PL, Seydoux G, Subramaniam K. Translational repression restricts expression of the C. elegans Nanos homolog NOS-2 to the embryonic germline. Dev Biol. 2006;292(1):244–52. doi: 10.1016/j.ydbio.2005.11.046 16499902

[45] Cao J, Packer JS, Ramani V, Cusanovich DA, Huynh C, Daza R, et al. Comprehensive single-cell transcriptional profiling of a multicellular organism. Science. 2017;357(6352):661–7. doi: 10.1126/science.aam8940 28818938 PMC5894354

[46] Sijen T, Fleenor J, Simmer F, Thijssen KL, Parrish S, Timmons L, et al. On the role of RNA amplification in dsRNA-triggered gene silencing. Cell. 2001;107(4):465–76. doi: 10.1016/s0092-8674(01)00576-1 11719187

[47] Kumsta C, Hansen M. C. elegans rrf-1 Mutations Maintain RNAi Efficiency in the Soma in Addition to the Germline. PLoS One. 2012;7. doi: 10.1371/journal.pone.0035428 22574120 PMC3344830

[48] Tijsterman M, Okihara KL, Thijssen K, Plasterk RH. PPW-1, a PAZ/PIWI protein required for efficient germline RNAi, is defective in a natural isolate of C. elegans. Curr Biol. 2002;12(17):1535–40. doi: 10.1016/s0960-9822(02)01110-7 12225671

[49] Butuci M, Williams AB, Wong MM, Kramer B, Michael WM. Zygotic Genome Activation Triggers Chromosome Damage and Checkpoint Signaling in C. elegans Primordial Germ Cells. Dev Cell. 2015;34(1):85–95. doi: 10.1016/j.devcel.2015.04.019 26073019

[50] Checchi PM, Kelly WG. emb-4 is a conserved gene required for efficient germline-specific chromatin remodeling during Caenorhabditis elegans embryogenesis. Genetics. 2006;174(4):1895–906. doi: 10.1534/genetics.106.063701 17028322 PMC1698644

[51] Furuhashi H, Takasaki T, Rechtsteiner A, Li T, Kimura H, Checchi PM, et al. Trans-generational epigenetic regulation of C. elegans primordial germ cells. Epigenetics Chromatin. 2010;3(1):15. doi: 10.1186/1756-8935-3-15 20704745 PMC3146070

[52] Schaner CE, Deshpande G, Schedl PD, Kelly WG. A conserved chromatin architecture marks and maintains the restricted germ cell lineage in worms and flies. Dev Cell. 2003;5(5):747–57. doi: 10.1016/s1534-5807(03)00327-7 14602075 PMC4100483

[53] Chafin DR, Guo H, Price DH. Action of alpha-amanitin during pyrophosphorolysis and elongation by RNA polymerase II. J Biol Chem. 1995;270(32):19114–9. doi: 10.1074/jbc.270.32.19114 7642577

[54] Rudd MD, Luse DS. Amanitin greatly reduces the rate of transcription by RNA polymerase II ternary complexes but fails to inhibit some transcript cleavage modes. J Biol Chem. 1996;271(35):21549–58. doi: 10.1074/jbc.271.35.21549 8702941

[55] Wong MM, Belew MD, Kwieraga A, Nhan JD, Michael WM. Programmed DNA Breaks Activate the Germline Genome in Caenorhabditis elegans. Dev Cell. 2018;46(3):302–15 e5. doi: 10.1016/j.devcel.2018.07.002 30086301

[56] Gruidl ME, Smith PA, Kuznicki KA, McCrone JS, Kirchner J, Roussell DL, et al. Multiple potential germ-line helicases are components of the germ-line-specific P granules of Caenorhabditis elegans. Proc Natl Acad Sci U S A. 1996;93(24):13837–42. doi: 10.1073/pnas.93.24.13837 8943022 PMC19442

[57] Andralojc KM, Campbell AC, Kelly AL, Terrey M, Tanner PC, Gans IM, et al. ELLI-1, a novel germline protein, modulates RNAi activity and P-granule accumulation in Caenorhabditis elegans. PLoS Genet. 2017;13(2):e1006611. doi: 10.1371/journal.pgen.1006611 28182654 PMC5325599

[58] Spencer WC, Zeller G, Watson JD, Henz SR, Watkins KL, McWhirter RD, et al. A spatial and temporal map of C. elegans gene expression. Genome Res. 2011;21(2):325–41. doi: 10.1101/gr.114595.110 21177967 PMC3032935

[59] Ciosk R, DePalma M, Priess JR. Translational regulators maintain totipotency in the Caenorhabditis elegans germline. Science. 2006;311(5762):851–3. doi: 10.1126/science.1122491 16469927

[60] Mainpal R, Nance J, Yanowitz JL. A germ cell determinant reveals parallel pathways for germ line development in Caenorhabditis elegans. Development. 2015;142(20):3571–82. doi: 10.1242/dev.125732 26395476 PMC6514396

[61] Tursun B, Patel T, Kratsios P, Hobert O. Direct conversion of C. elegans germ cells into specific neuron types. Science. 2011;331(6015):304–8. doi: 10.1126/science.1199082 21148348 PMC3250927

[62] Updike DL, Knutson AK, Egelhofer TA, Campbell AC, Strome S. Germ-granule components prevent somatic development in the C. elegans germline. Curr Biol. 2014;24(9):970–5. doi: 10.1016/j.cub.2014.03.015 24746798 PMC4036631

[63] Angeles-Albores D, NL RY, Chan J, Sternberg PW. Tissue enrichment analysis for C. elegans genomics. BMC Bioinformatics. 2016;17(1):366. doi: 10.1186/s12859-016-1229-9 27618863 PMC5020436

[64] Angeles-Albores D, Sternberg PW. Using Transcriptomes as Mutant Phenotypes Reveals Functional Regions of a Mediator Subunit in Caenorhabditis elegans. Genetics. 2018;210(1):15–24. doi: 10.1534/genetics.118.301133 30030292 PMC6116950

[65] Lee CS, Lu T, Seydoux G. Nanos promotes epigenetic reprograming of the germline by down-regulation of the THAP transcription factor LIN-15B. Elife. 2017;6. doi: 10.7554/eLife.30201 29111977 PMC5734877

[66] Baugh LR, Demodena J, Sternberg PW. RNA Pol II accumulates at promoters of growth genes during developmental arrest. Science. 2009;324(5923):92–4. doi: 10.1126/science.1169628 19251593

[67] Hutter H, Suh J. GExplore 1.4: An expanded web interface for queries on Caenorhabditis elegans protein and gene function. Worm. 2016;5(4):e1234659. doi: 10.1080/21624054.2016.1234659 28090394 PMC5190144

[68] Long X, Spycher C, Han ZS, Rose AM, Muller F, Avruch J. TOR deficiency in C. elegans causes developmental arrest and intestinal atrophy by inhibition of mRNA translation. Curr Biol. 2002;12(17):1448–61. doi: 10.1016/s0960-9822(02)01091-6 12225660

[69] Webster AK, Chitrakar R, Baugh LR. Alternative somatic and germline gene-regulatory strategies during starvation-induced developmental arrest. BioRxiv [Preprint]. doi: 10.1101/2021.04.30.441514 PMC9608353 36223742

[70] Subramaniam K, Seydoux G. nos-1 and nos-2, two genes related to Drosophila nanos, regulate primordial germ cell development and survival in Caenorhabditis elegans. Development. 1999;126(21):4861–71. 10518502 10.1242/dev.126.21.4861

[71] McIntyre DC, Nance J. Niche Cell Wrapping Ensures Primordial Germ Cell Quiescence and Protection from Intercellular Cannibalism. Curr Biol. 2020;30(4):708–14 e4. doi: 10.1016/j.cub.2019.12.021 32008902 PMC7044025

[72] Demoinet E, Li S, Roy R. AMPK blocks starvation-inducible transgenerational defects in Caenorhabditis elegans. Proc Natl Acad Sci U S A. 2017;114(13):E2689–E98. doi: 10.1073/pnas.1616171114 28289190 PMC5380097

[73] Yang W, Dierking K, Schulenburg H. WormExp: a web-based application for a Caenorhabditis elegans-specific gene expression enrichment analysis. Bioinformatics. 2016;32(6):943–5. doi: 10.1093/bioinformatics/btv667 26559506

[74] Chang HW, Pisano S, Chaturbedi A, Chen J, Gordon S, Baruah A, et al. Transcription factors CEP-1/p53 and CEH-23 collaborate with AAK-2/AMPK to modulate longevity in Caenorhabditis elegans. Aging Cell. 2017;16(4):814–24. doi: 10.1111/acel.12619 28560849 PMC5506430

[75] Steinbach N, Hasson D, Mathur D, Stratikopoulos EE, Sachidanandam R, Bernstein E, et al. PTEN interacts with the transcription machinery on chromatin and regulates RNA polymerase II-mediated transcription. Nucleic Acids Res. 2019;47(11):5573–86. doi: 10.1093/nar/gkz272 31169889 PMC6582409

[76] Chen ZH, Zhu M, Yang J, Liang H, He J, He S, et al. PTEN interacts with histone H1 and controls chromatin condensation. Cell Rep. 2014;8(6):2003–14. doi: 10.1016/j.celrep.2014.08.008 25199838 PMC4201947

[77] Gong L, Govan JM, Evans EB, Dai H, Wang E, Lee SW, et al. Nuclear PTEN tumor-suppressor functions through maintaining heterochromatin structure. Cell Cycle. 2015;14(14):2323–32. doi: 10.1080/15384101.2015.1044174 25946202 PMC4614552

[78] Swygert SG, Kim S, Wu X, Fu T, Hsieh TH, Rando OJ, et al. Condensin-Dependent Chromatin Compaction Represses Transcription Globally during Quiescence. Mol Cell. 2019;73(3):533–46 e4. doi: 10.1016/j.molcel.2018.11.020 30595435 PMC6368455

[79] Fields BD, Kennedy S. Chromatin Compaction by Small RNAs and the Nuclear RNAi Machinery in C. elegans. Sci Rep. 2019;9(1):9030. doi: 10.1038/s41598-019-45052-y 31227740 PMC6588724

[80] Weiser NE, Yang DX, Feng S, Kalinava N, Brown KC, Khanikar J, et al. MORC-1 Integrates Nuclear RNAi and Transgenerational Chromatin Architecture to Promote Germline Immortality. Dev Cell. 2017;41(4):408–23 e7. doi: 10.1016/j.devcel.2017.04.023 28535375 PMC5527976

[81] Stiernagle T. Maintenance of C. elegans 2006, In: *WormBook*, ed. The *C*. *elegans* Research Community, WormBook, doi: 10.1895/wormbook.1.101.1, http://www.wormbook.org PMC4781397 18050451

[82] Evans TC. Transformation and microinjection, (2006), *WormBook*, ed. The *C*. *elegans* Research Community, WormBook, doi: 10.1895/wormbook.1.108.1,http://www.wormbook.org.

[83] Brandt JP, Ringstad N. Toll-like Receptor Signaling Promotes Development and Function of Sensory Neurons Required for a C. elegans Pathogen-Avoidance Behavior. Curr Biol. 2015;25(17):2228–37. doi: 10.1016/j.cub.2015.07.037 26279230 PMC4642686

[84] Dokshin GA, Ghanta KS, Piscopo KM, Mello CC. Robust Genome Editing with Short Single-Stranded and Long, Partially Single-Stranded DNA Donors in Caenorhabditis elegans. Genetics. 2018;210(3):781–7. doi: 10.1534/genetics.118.301532 30213854 PMC6218216

[85] Timmons L, Court DL, Fire A. Ingestion of bacterially expressed dsRNAs can produce specific and potent genetic interference in Caenorhabditis elegans. Gene. 2001;263(1–2):103–12. doi: 10.1016/s0378-1119(00)00579-5 11223248

[86] Zhang S, Kuhn J. Cell isolation and culture. (2013), *WormBook*, ed. The *C*. *elegans* Research Community, WormBook, doi: 10.1895/wormbook.1.157.1, http://www.wormbook.org. PMC4781291 23430760

[87] Seydoux G, Dunn MA. Transcriptionally repressed germ cells lack a subpopulation of phosphorylated RNA polymerase II in early embryos of Caenorhabditis elegans and Drosophila melanogaster. Development. 1997;124(11):2191–201. 9187145 10.1242/dev.124.11.2191

[88] Bowman EA, Bowman CR, Ahn JH, Kelly WG. Phosphorylation of RNA polymerase II is independent of P-TEFb in the C. elegans germline. Development. 2013;140(17):3703–13. doi: 10.1242/dev.095778 23903194 PMC3742150

[89] Wang S, Fisher K, Poulin GB. Lineage specific trimethylation of H3 on lysine 4 during C. elegans early embryogenesis. Dev Biol. 2011;355(2):227–38. doi: 10.1016/j.ydbio.2011.04.010 21549110

[90] Xiao Y, Bedet C, Robert VJ, Simonet T, Dunkelbarger S, Rakotomalala C, et al. Caenorhabditis elegans chromatin-associated proteins SET-2 and ASH-2 are differentially required for histone H3 Lys 4 methylation in embryos and adult germ cells. Proc Natl Acad Sci U S A. 2011;108(20):8305–10. doi: 10.1073/pnas.1019290108 21527717 PMC3100940

[91] Shakes DC, Miller DM 3rd, Nonet ML. Immunofluorescence microscopy. Methods Cell Biol. 2012;107:35–66. doi: 10.1016/B978-0-12-394620-1.00002-3 22226520

[92] Kamath RS, Fraser AG, Dong Y, Poulin G, Durbin R, Gotta M, et al. Systematic functional analysis of the Caenorhabditis elegans genome using RNAi. Nature. 2003;421(6920):231–7. doi: 10.1038/nature01278 12529635

[93] Langmead B, Trapnell C, Pop M, Salzberg SL. Ultrafast and memory-efficient alignment of short DNA sequences to the human genome. Genome Biol. 2009;10(3):R25. doi: 10.1186/gb-2009-10-3-r25 19261174 PMC2690996

[94] Anders S, Pyl PT, Huber W. HTSeq—a Python framework to work with high-throughput sequencing data. Bioinformatics. 2015;31(2):166–9. doi: 10.1093/bioinformatics/btu638 25260700 PMC4287950

[95] Robinson MD, McCarthy DJ, Smyth GK. edgeR: a Bioconductor package for differential expression analysis of digital gene expression data. Bioinformatics. 2010;26(1):139–40. doi: 10.1093/bioinformatics/btp616 19910308 PMC2796818

[96] Hunt-Newbury R, Viveiros R, Johnsen R, Mah A, Anastas D, Fang L, et al. High-throughput in vivo analysis of gene expression in Caenorhabditis elegans. PLoS Biol. 2007;5(9):e237. doi: 10.1371/journal.pbio.0050237 17850180 PMC1971126

