# Supplementary figures and images for: DAF-18/PTEN inhibits germline zygotic gene activation during primordial germ cell quiescence
Source: PLoS Genet. 2021 Jul 21;17(7):e1009650. doi: 10.1371/journal.pgen.1009650 (PMC8294487; doi:10.1371/journal.pgen.1009650)

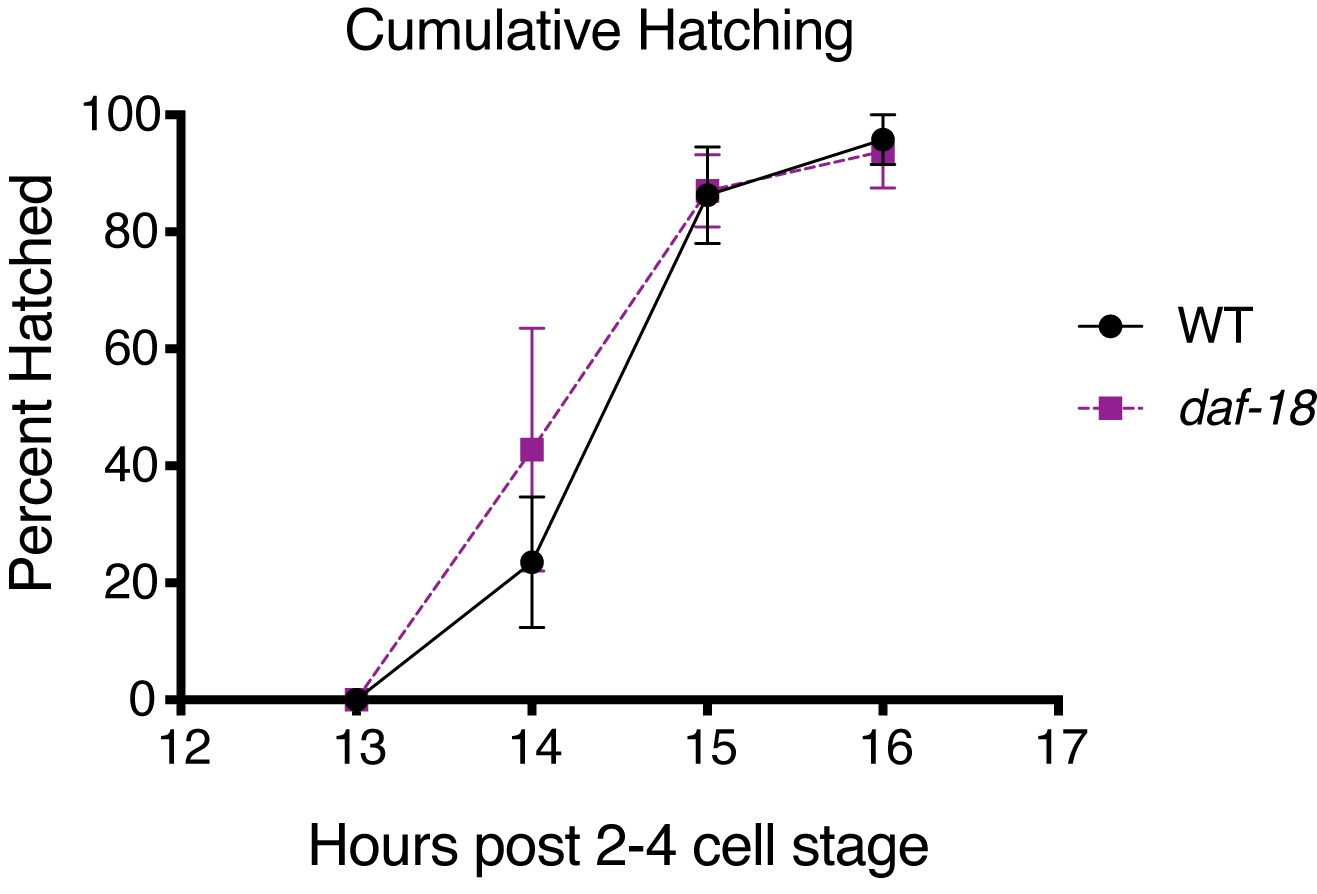

Supplement: S1 Fig — The number of L1 larvae hatched from embryos isolated at the 2–4 cell stage was monitored hourly. 4 replicate experiments (2 biological, 2 technical), containing a total of 40 WT (PGC::mCherry, naSi2) animals, and 49 daf-18 mutant animals (naSi2; daf-18). (TIF) [file pgen.1009650.s001.tif]

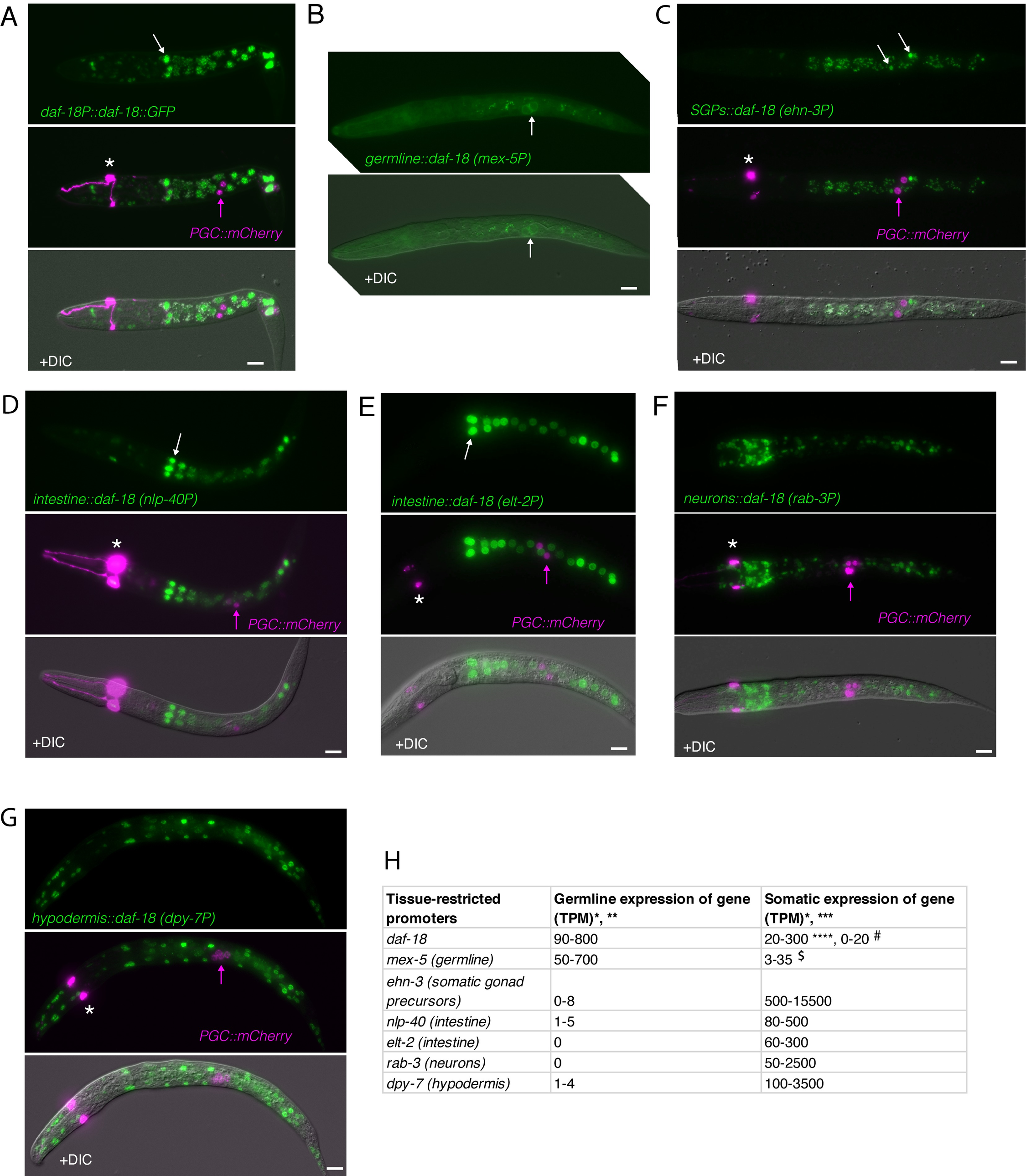

Supplement: S2 Fig — (A-G) All animals are daf-18(ok480) mutants, and all carry PGC::mCherry (naSi2), except for panel (B). Scale bars represent 10μm, white arrows indicate GFP expression in expected tissues-of-interest, pink arrows indicate PGCs, and white stars indicate a transgenic co-injection marker (flp-17P::dsRed) which was used to identify all extrachromosomal array-bearing animals. All daf-18(+) constructs (various promoters driving daf-18(+)::SL2::GFP::H2B) are carried as extrachromosomal arrays (with nuclear GFP for cell identification), except for germline::daf-18(+) (in panel (B)), which is a single copy insertion. Arrays carry the daf-18 3’UTR on the daf-18 rescuing portion, and the unc-54 3’UTR for the GFP reporter portion to allow broad somatic expression [96] (e.g. rab-3P::daf-18(+)::daf-18 3’UTR::SL2::GFP::H2B::unc-54 3’UTR). (B) The germline:: daf-18(+) construct (mex-5P::SV40 NLS::daf-18 coding region::nos-2 3’UTR::SL2::GFPo::PH::tbb-2 3’UTR) has several features. First, the GFP is fused to a PH domain thereby targeting the GFP to the cell membrane. Second, there are two different 3’ UTRs, one following the daf-18 portion and another following the GFP. Specifically, the nos-2 3’UTR follows the daf-18 rescuing portion (as used in our PGC::mCherry, seen in the other panels of this figure), which restricts expression to the PGCs (1 additional head cell is marked in ~10% of animals). The tbb-2 3’UTR was used for the GFP portion, which allows somewhat broader expression as is visible in somatic tissues. Nevertheless, daf-18(+) expression should be limited to PGCs, as evidenced by the PGC::mCherry reporter naSi2 which expresses mCherry from the exact same mex-5 promoter and the same nos-2 3’ sequence. Last, an NLS is fused in frame with DAF-18; it is possible that it enhances DAF-18 nuclear activity. (H) Approximate embryonic transcript levels [41], in germline and somatic tissues of interest, of genes for which promoters were used to drive tissue-specific expression. *Adj [file pgen.1009650.s002.tif]

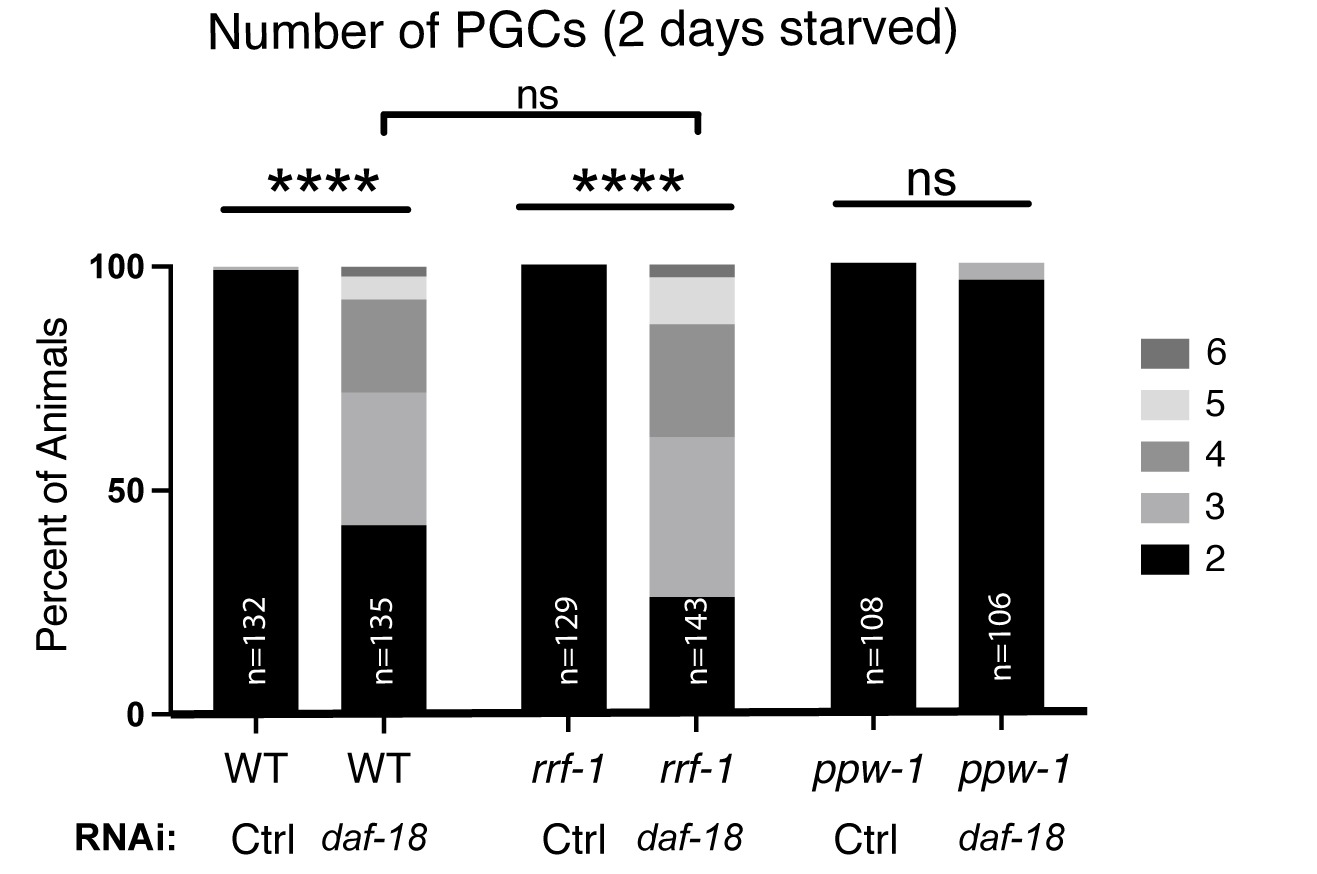

Supplement: S3 Fig — F3 progeny of animals fed HT115 bacteria expressing control (Ctrl), empty vector L4440, or daf-18 dsRNA (feeding from P0 to F2) were assessed for number of PGCs after 2 days of starvation as L1s. All strains carry naSi2 (PGC::mCherry). n values display the total number of L1 larvae examined for each genotype across three biological replicates in which all three strains were subject to RNAi in parallel in each replicate. Two-sided Fisher’s exact tests were performed to compare within genotype and statistical significance is displayed (ns p>0.05, ****p<0.0001). (TIF) [file pgen.1009650.s003.tif]

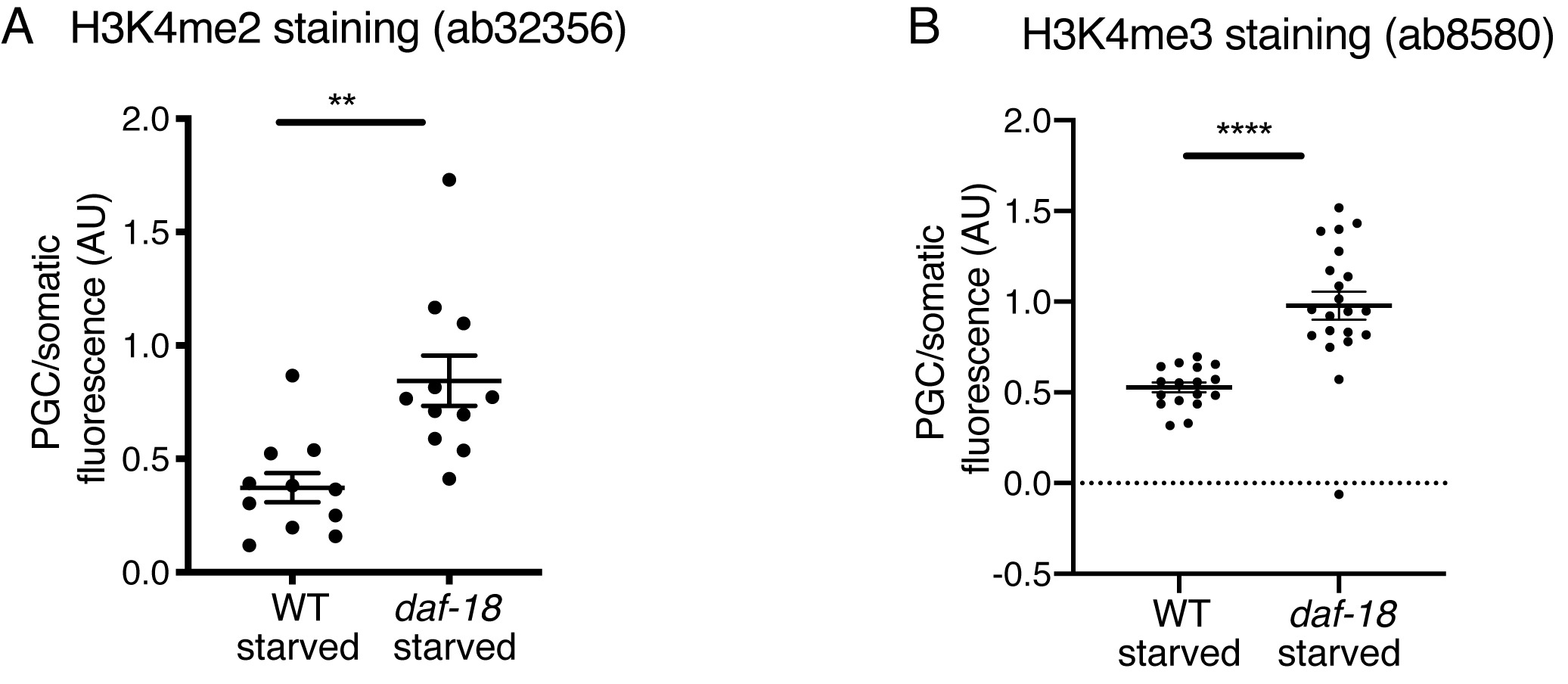

Supplement: S4 Fig — (A,B). Mean staining fluorescence was measured for each PGC nucleus and its nearest somatic cell nucleus (from single image slices), and is displayed in the graph as a ratio. (A) Antibody ab32356 (H3K4me2) was used to stain wild type (WT) and daf-18(ok480) mutant L1s, starved 6–8 hours after hatching (synchronized within 2 hours of hatching and imaged 6 hours later). PGCs were identified with anti-PGL-1 antibody (OIC1D4). Each dot represents a single PGC/somatic cell pair (sometimes 2 values per animal). (B) Antibody ab8580 (H3K4me3) was used to stain WT and daf-18(ok480) mutant L1s hatched and starved overnight (~16h). PGCs were identified with encoded glh-1::GFP. Each dot represents one value per worm (2 PGC/somatic values averaged). Statistical significance determined by two-tailed T-test. **p<0.01, ****p<0.0001. (TIF) [file pgen.1009650.s004.tif]

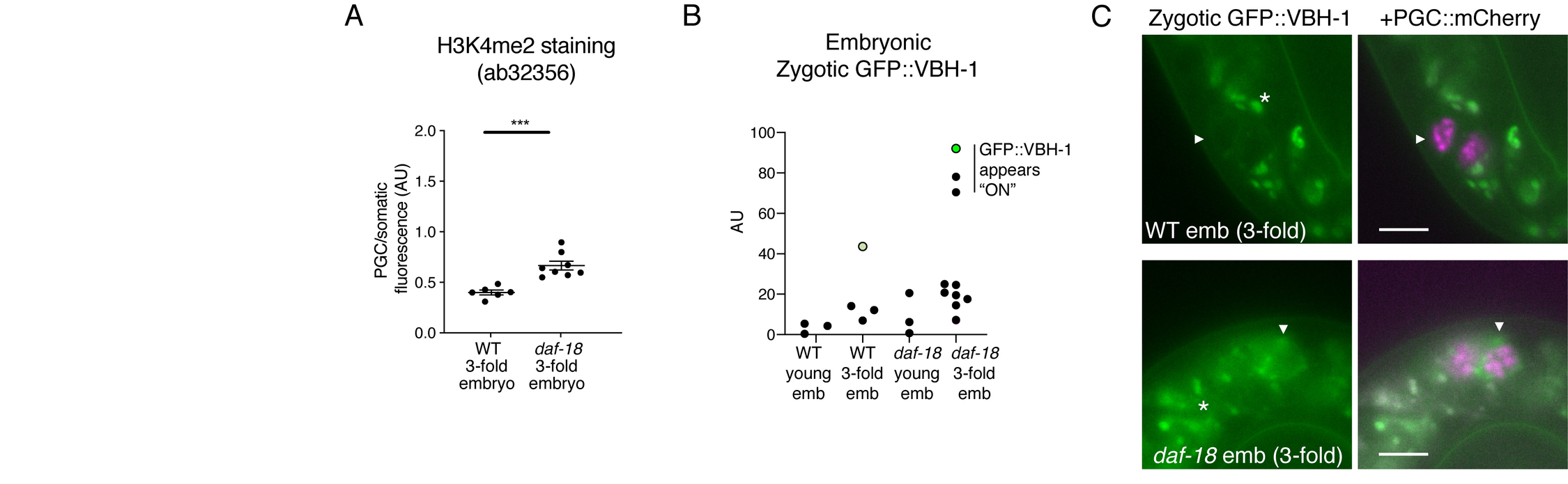

Supplement: S5 Fig — (A) Antibody ab32356 (anti-H3K4me2) was used to stain wild type (WT) and daf-18(ok480) mutant 3-fold embryos. PGCs were identified with glh-1::GFP. Mean fluorescence was measured for each PGC and its nearest somatic cell (from single image slices), and is displayed in the graph as a ratio. Each dot is a single PGC/somatic cell pair. Statistical significance determined by two-tailed T-test. ***p<0.001. (B) and (C) Zygotic expression of GFP::VBH-1 was examined by epifluorescence (as in Fig 4) in embryonic PGCs. (B) Mean perinuclear GFP fluorescence intensity (AU = Arbitrary Units) values are plotted. Graph shows results from three separate experiments pooled. Each dot represents one animal; colored dots correspond to images in panel (C). (C) Images of the brightest perinuclear GFP::VBH-1, in PGCs of WT and daf-18 mutants, according to quantification. Each arrowhead points to perinuclear GFP of one PGC. Gut granules (green autofluorescent dots) are marked with asterisks. Scale bars represent 10μm. (TIF) [file pgen.1009650.s005.tif]

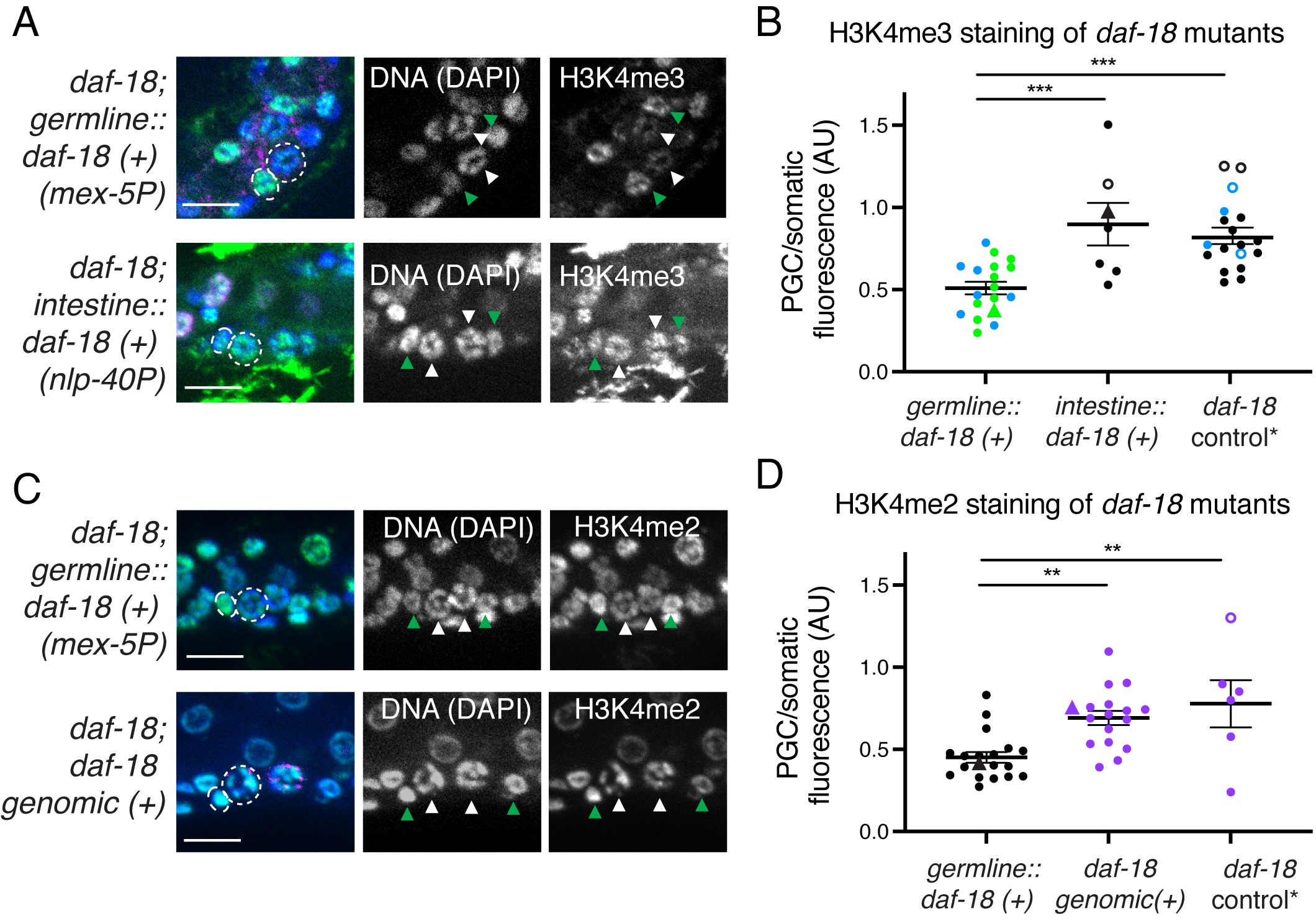

Supplement: S6 Fig — Antibodies ab8580 (anti-H3K4me3) (A, B) and CMA303 (anti-H3K4me2) (C, D) were used to stain L1 larvae starved for up to 24 hours. Tricolor images (left panels) show DNA (DAPI/blue), histone mark immunofluorescence (green), and either a PGC marker (see below) or transgenic animal marker (magenta). Maximum intensity projections of image stacks are shown, except for (A) (top panels), which show a single slice. Dashed circle indicates one PGC in each image, and dashed smaller ellipse indicates its nearest somatic cell (likely its SGP). White arrowheads point to PGCs, and green arrowheads point to neighboring somatic cells. Scale bars represent 10μm. Mean staining fluorescence was quantified for each PGC nucleus and its nearest somatic cell nucleus (from single image slices), and is displayed in the correlated graph as a ratio. Each dot in the graph represents one animal (2 PGC/SGP values averaged or a single PGC/SGP pair). Dot colors represent data gathered on different days / in different experiments. Triangles represent animals shown in the images, and open circles represent animals with 3 or more PGCs. Statistical significance determined by one-way ANOVA. **p<0.01, ***p<0.001. (A) Magenta marks germ cell membranes (anti-GFP in L1 larvae carrying germline::daf-18::SL2::GFP::PH) (top panels) or intestinal cells (anti-GFP in L1s carrying intestine::daf-18::SL2::GFP::H2B) (bottom panels). PGCs were identified by anti-GFP membrane marker (top) or DIC (bottom). Transgenic animals carrying daf-18(+) rescuing arrays were identified by anti-GFP. (B) “daf-18 control*” represents non-transgenic siblings of daf-18; intestine::daf-18(+) animals in the middle column (black dots), and a mix of transgenic and non-transgenic daf-18; neurons::daf-18(+) (blue dots). Since these are F3 progeny of P0 mothers that were selected (transgene-positive), possible maternal contributions of transgenes are unknown.(C) Magenta marks germ cell P granules (glh-1::GFP, falsely colored, bottom panels) [file pgen.1009650.s006.tif]

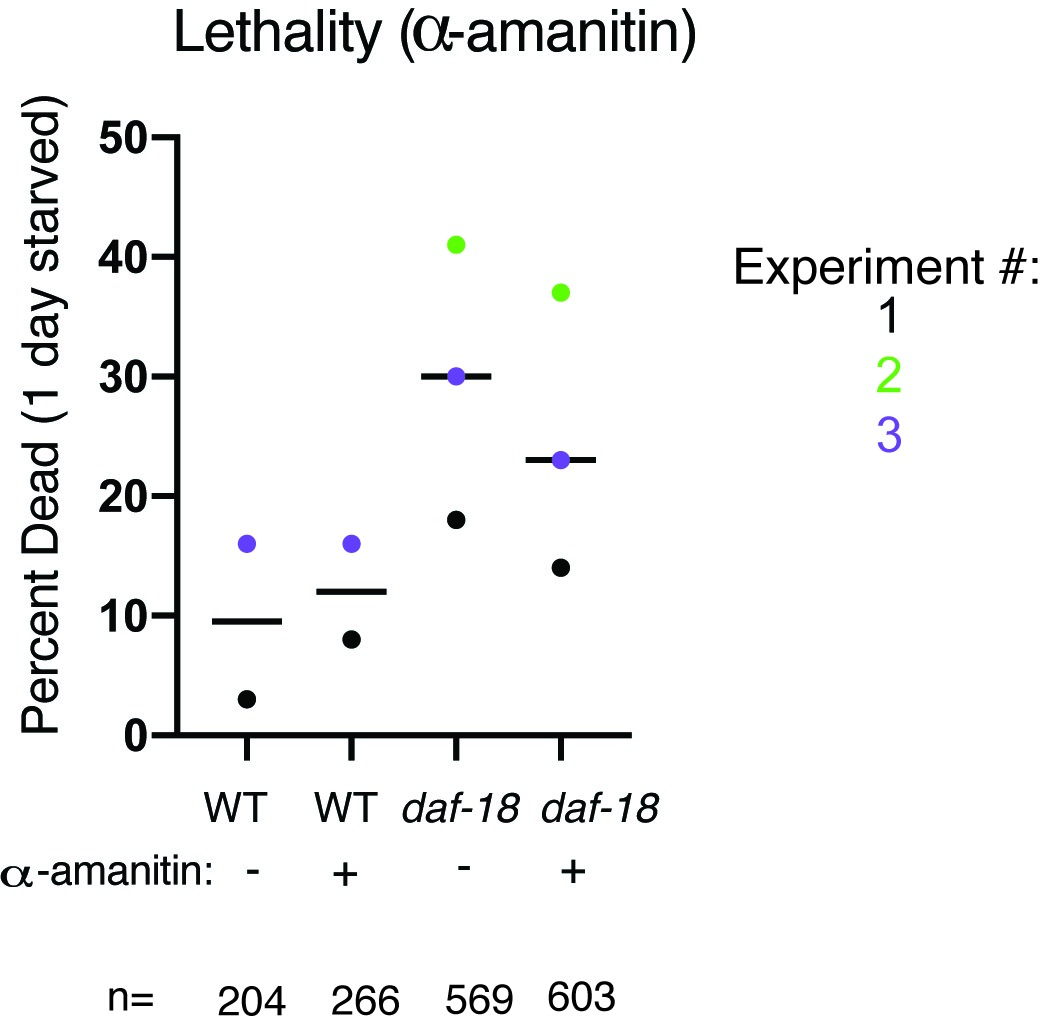

Supplement: S7 Fig — Embryos (from a bleach preparation) carrying a PGC marker (naSi2 PGC::mCherry or sam24 glh-1::GFP) were allowed to hatch and starve up to 24h, with or without 10ug/mL α-amanitin, before assessing PGC numbers (see Fig 3E) and dead/total L1 larvae. Colors represent different replicates. Line indicates the median. (TIF) [file pgen.1009650.s007.tif]

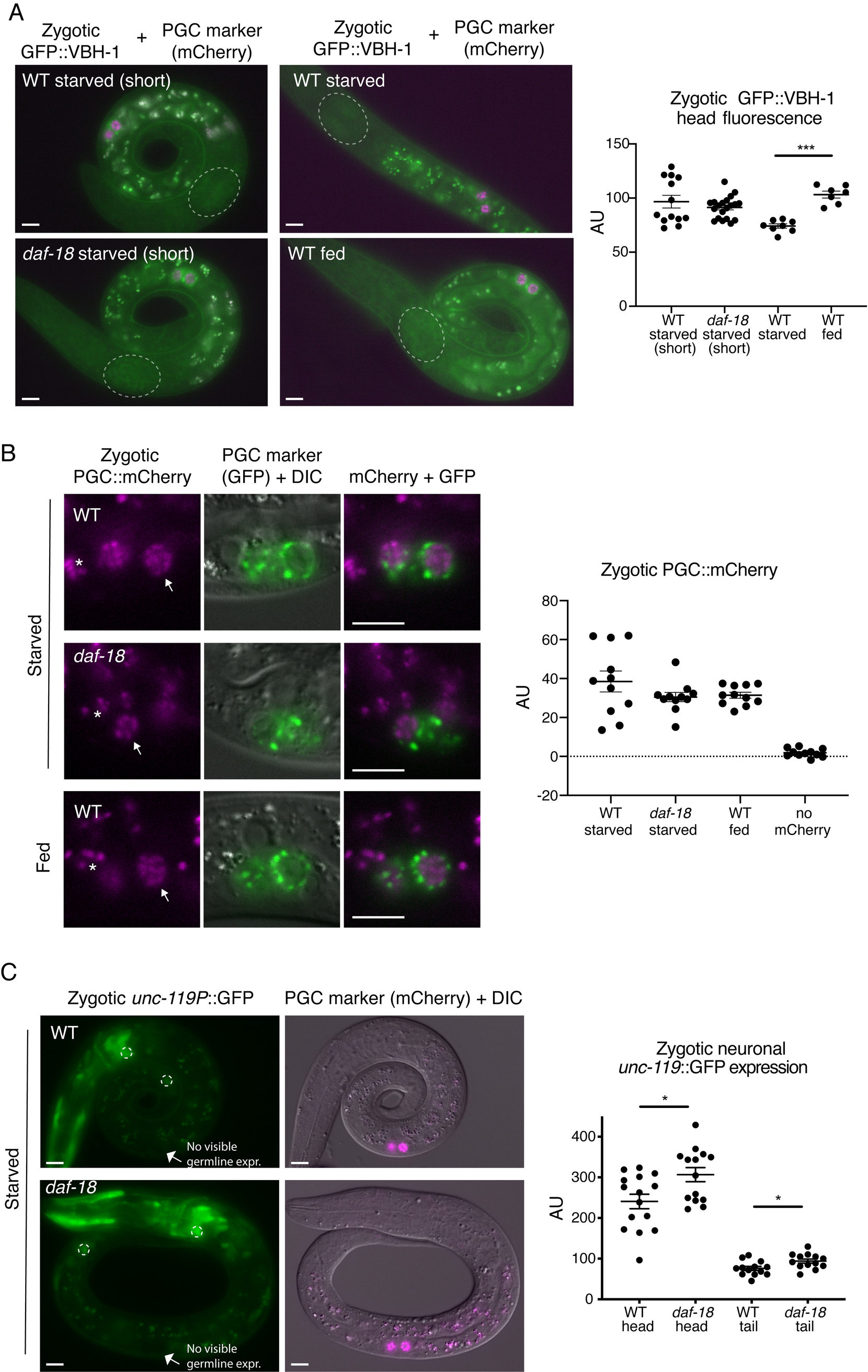

Supplement: S8 Fig — (A-C) Images show epifluorescence. Mean fluorescence intensity (AU = Arbitrary Units) values are plotted to the right of each set of images. Each dot represents one animal. Non-cross progeny were identified by the lack of somatic GFP ((A) and (C)) or lack of PGC::mCherry (B), which are all visible when present, even in starved animals. Mean +/- SEM. All scale bars represent 10μm. (A) Zygotic GFP::VBH-1 expression in the soma (head neurons) is not expressed more highly in starved daf-18 mutants than starved wild type (WT). Dashed ellipses show regions measured for mean fluorescence. As in Fig 4, “Starved (short)” animals were imaged within 5 hours of hatching. “Starved” progeny were imaged up to 22 hours after clean embryo preparation, while “fed” L1 progeny were imaged at the same time, but after 5 hours of feeding (after up to 17 hours post-embryo preparation). Graph shows the pooled results of 2 experiments. Statistical significance determined by one-way ANOVA with Tukey’s multiple comparisons test. ***p<0.001. (B) Zygotic nuclear PGC::mCherry expression is not expressed more highly in starved daf-18 mutant PGCs than starved WT PGCs. L1s were imaged up to 19 hours after clean embryo preparation. Arrows point to zygotic PGC::mCherry expression in PGCs, and asterisks mark auto-fluorescent gut granules. Nuclear PGC::mCherry fluorescence was measured by hand-drawing regions of interest around PGC nuclei. One experimental replicate is graphed, and 2 others showed similar results. Statistical significance determined by one-way ANOVA with Tukey’s multiple comparisons test. (C) Zygotic unc-119P::GFP is not expressed in the PGCs of starved daf-18 mutants. However, it is expressed at a slightly higher level in neurons of starved daf-18 mutants. L1s were imaged up to 18 hours after clean embryo preparation. Dashed circles indicate approximate neuronal regions measured (one head neuron and one tail neuron per animal). Graph shows the pooled results of 2 experiments. Statistic [file pgen.1009650.s008.tif]

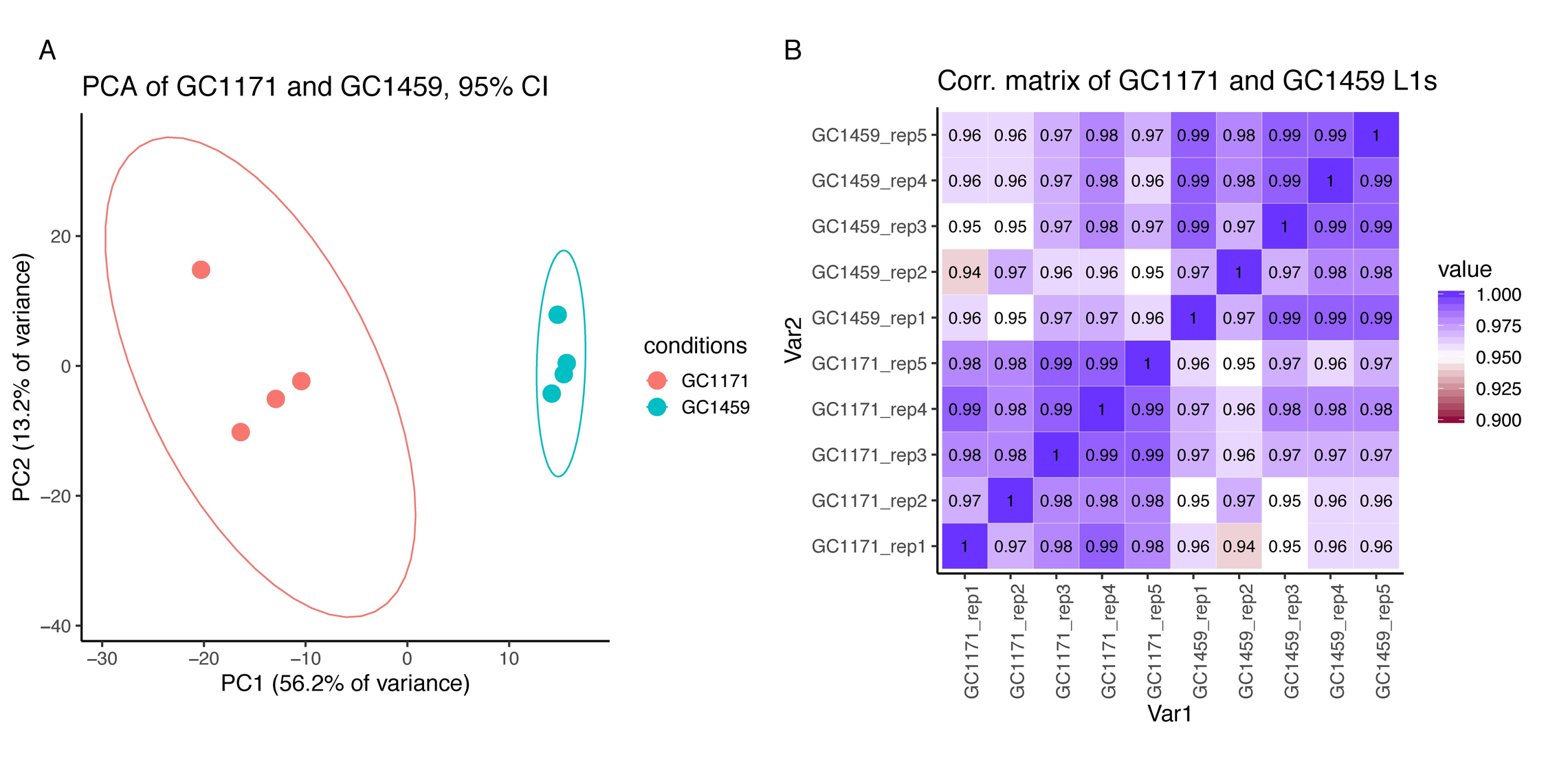

Supplement: S9 Fig — (A) Principal component analysis (PCA) of GC1171 (wild type) and GC1459 (daf-18(ok480)) starved L1s in RNA Sequencing experiment, highlighting low variance within each genotype and substantial variance between genotypes. (B) Correlation matrix of GC1171 (wild type) and GC1459 (daf-18(ok480)) showing that replicates of the same genotype were highly correlated. (TIF) [file pgen.1009650.s009.tif]

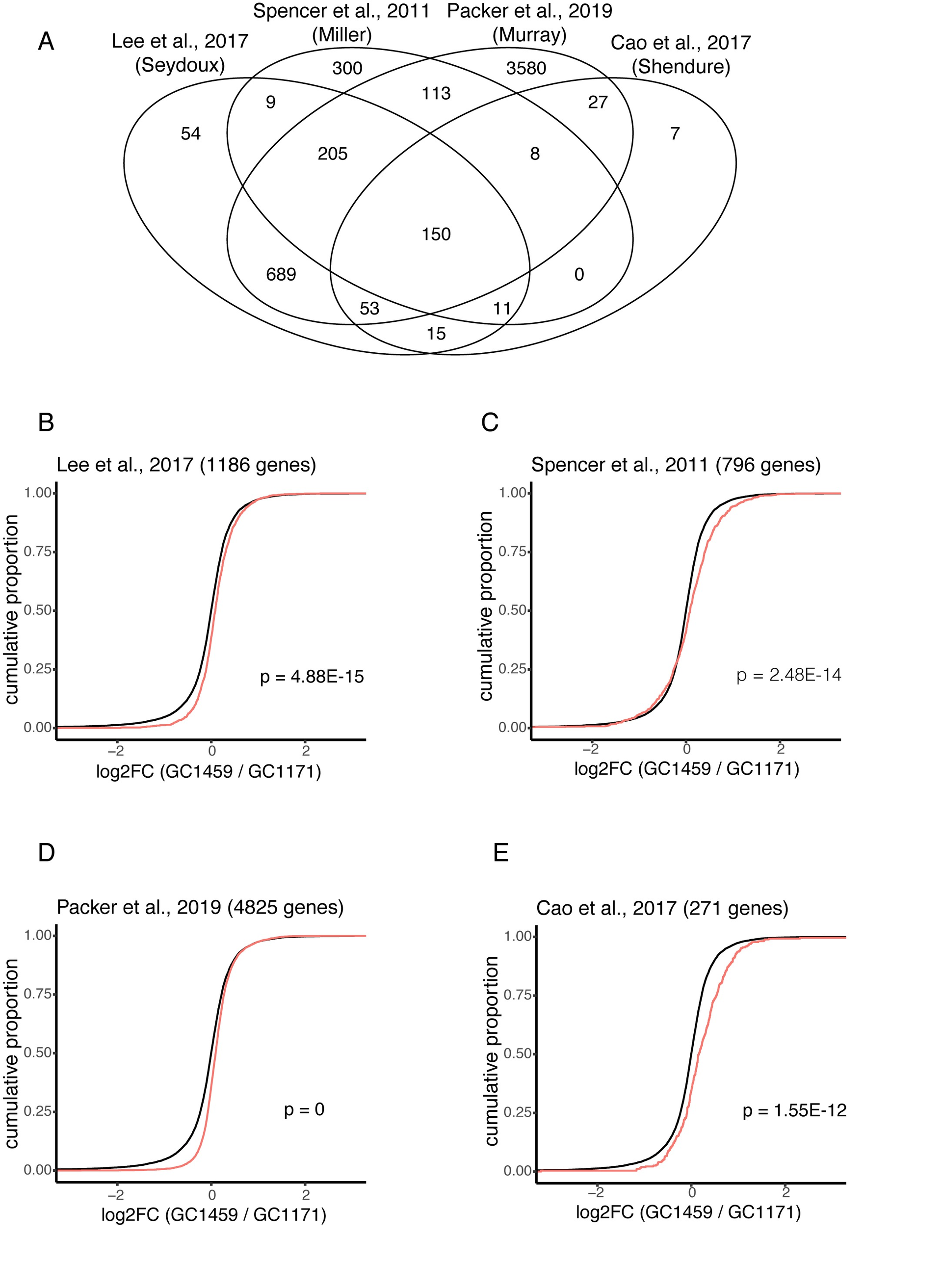

Supplement: S10 Fig — (A) Venn diagram showing the overlap of all four germline gene sets analyzed [41,45,58,65], and numbers of genes from these sets detected in our RNA Sequencing experiment. (B-E) Cumulative distribution function (CDF) plots for individual published germline gene sets (red lines) compared to the background set of all transcripts detected in our experiment (12,592 genes). p-values determined by Kolmogorov-Smirnov (KS) test are shown. Numbers of transcripts from published gene sets detected in our experiment are shown in graph titles (parentheses). (TIF) [file pgen.1009650.s010.tif]

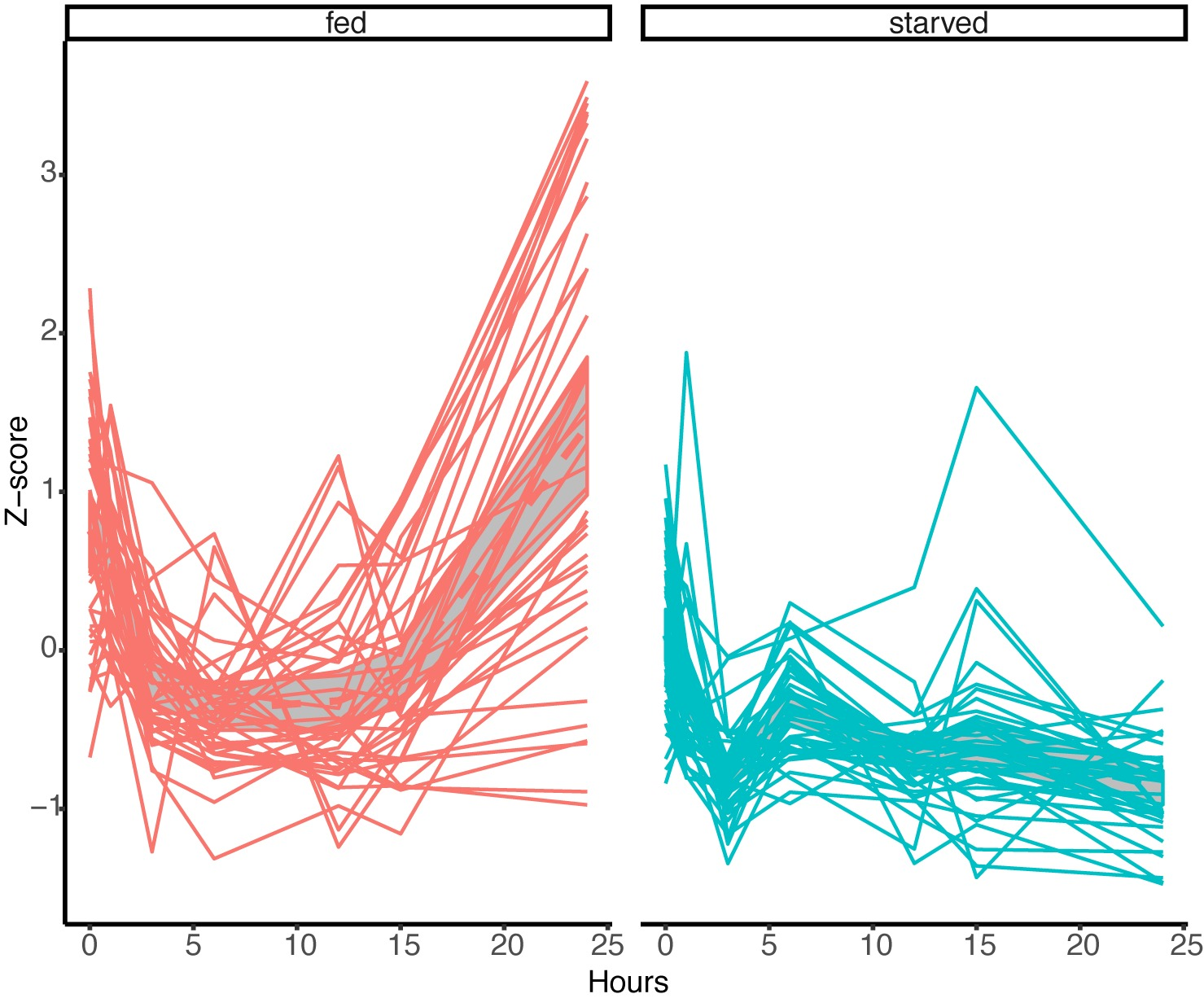

Supplement: S11 Fig — Individual Z-scores of these 40 genes (those detected in this dataset out of 42 identified in Fig 5E) are plotted over time, and confidence intervals (95%) of their average are plotted in grey. (TIF) [file pgen.1009650.s011.tif]

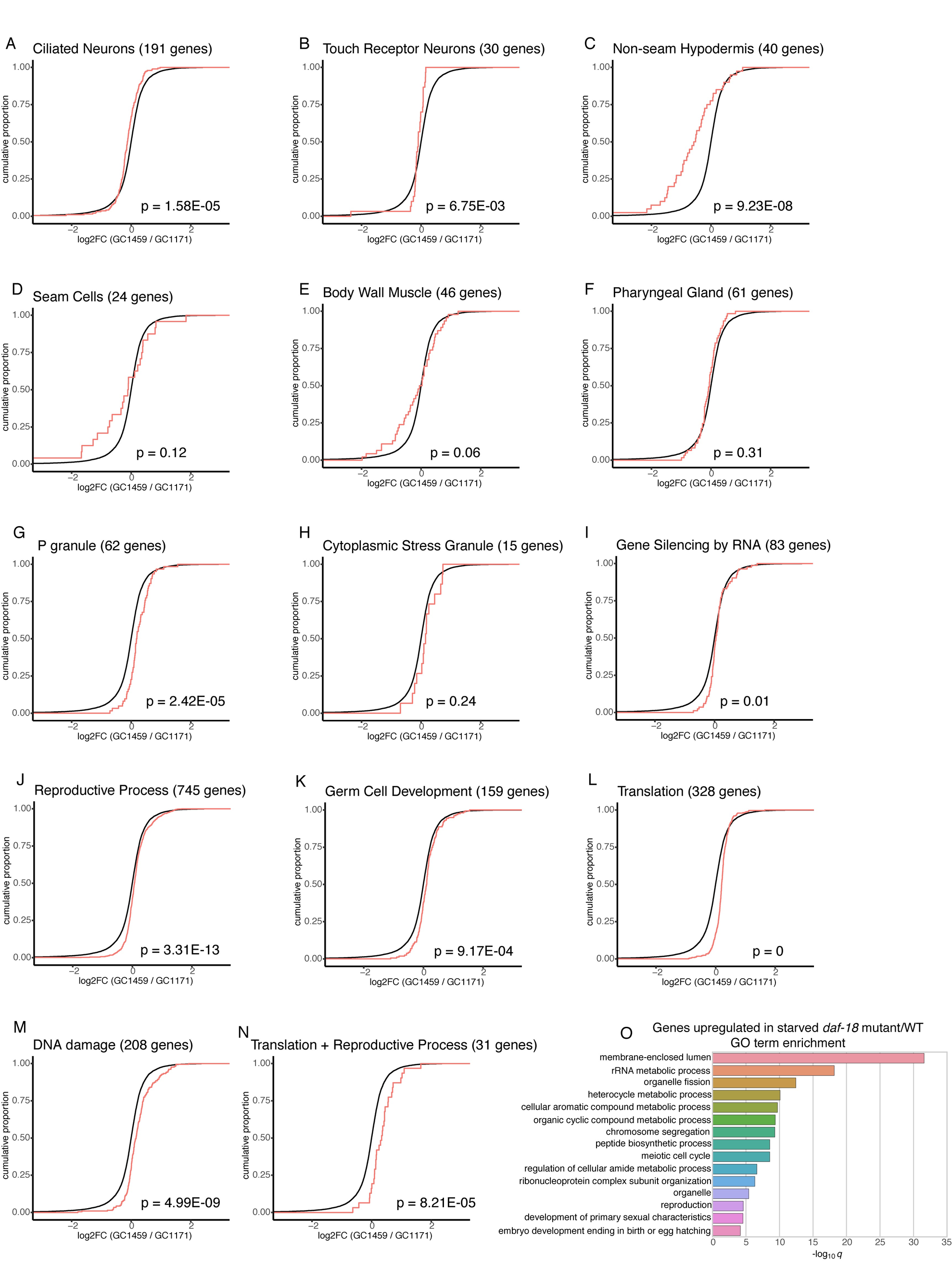

Supplement: S12 Fig — Cumulative distribution function (CDF) plots of transcript sets of interest (red line) versus the background set of all 12,592 transcripts detected (black line). p-values determined by Kolmogorov-Smirnov (KS) test are shown. Numbers of transcripts from published gene sets detected in our experiment are shown in graph titles (parentheses). (A-F) CDF plots for lists of transcripts from tissues of interest known to express daf-18 (ciliated neurons, non-seam hypodermis, seam cells, body wall muscle), and tissues that may not express daf-18 (touch receptor neurons, pharyngeal gland) [41,45]. Tissue-specific gene lists were generated using the GExplore1.4 web tool (underlying data from Cao et al., 2017). (G-N) CDF plots for lists of transcripts associated with specific gene ontology (GO) terms (using WormBase). (O) Unbiased analysis of gene ontology (GO) terms, using WormBase’s Enrichment Analysis Tool. (TIF) [file pgen.1009650.s012.tif]
